# Interleukins, Chemokines, and Tumor Necrosis Factor Superfamily Ligands in the Pathogenesis of West Nile Virus Infection

**DOI:** 10.3390/v15030806

**Published:** 2023-03-22

**Authors:** Emna Benzarti, Kristy O. Murray, Shannon E. Ronca

**Affiliations:** 1 Department of Pediatrics, Division of Tropical Medicine, Baylor College of Medicine and Texas Children’s Hospital, Houston, TX 77030, USA; 2 William T. Shearer Center for Human Immunobiology, Texas Children’s Hospital, Houston, TX 77030, USA; 3 National School of Tropical Medicine, Baylor College of Medicine, Houston, TX 77030, USA; 4 Department of Immunology and Microbiology, Baylor College of Medicine, Houston, TX 77030, USA; 5 Department of Molecular Virology and Microbiology, Baylor College of Medicine, Houston, TX 77030, USA

**Keywords:** West Nile virus, cytokines, interleukins, chemokines, tumor necrosis factor superfamily ligands, infection model

## Abstract

West Nile virus (WNV) is a mosquito-borne pathogen that can lead to encephalitis and death in susceptible hosts. Cytokines play a critical role in inflammation and immunity in response to WNV infection. Murine models provide evidence that some cytokines offer protection against acute WNV infection and assist with viral clearance, while others play a multifaceted role WNV neuropathogenesis and immune-mediated tissue damage. This article aims to provide an up-to-date review of cytokine expression patterns in human and experimental animal models of WNV infections. Here, we outline the interleukins, chemokines, and tumor necrosis factor superfamily ligands associated with WNV infection and pathogenesis and describe the complex roles they play in mediating both protection and pathology of the central nervous system during or after virus clearance. By understanding of the role of these cytokines during WNV neuroinvasive infection, we can develop treatment options aimed at modulating these immune molecules in order to reduce neuroinflammation and improve patient outcomes.

## 1. Introduction

West Nile virus (WNV) is a positive-sense, single-stranded RNA virus belonging to the Japanese encephalitis serocomplex, genus *Flavivirus*, family *Flaviviridae* [1]. Its life cycle mainly involves birds and mosquitoes, whereas humans, horses, and other vertebrates are considered incidental hosts [2]. The WNV genome is translated into a single polypeptide and co- and post-translationally processed into ten proteins: three structural (capsid C, membrane precursor prM, and envelope E), which form the virion; seven nonstructural proteins (NS1, NS2A, NS2B, NS3, NS4A, 2K, NS4B, and NS5) involved in the viral replication cycle, evasion of host innate immunity, and WNV pathogenesis [3]; and one peptide 2K, which plays a role in rearranging cytoplasmic membranes and Golgi trafficking of the NS4A protein [4]. The susceptibility to WNV is highly variable among its hosts [5]. The majority of WNV infections in humans are either asymptomatic or mild, presenting with headache, weakness, and/or fever [6]. However, a small percentage of WNV-infected patients (less than 1% [7]) will develop neuroinvasive disease, including meningitis, encephalitis, or acute flaccid paralysis, for whom death occurs in 10–30% of cases [8,9]. Long-term physical and neurocognitive sequelae, including weakness, fatigue, myalgia, memory or hearing loss, depression, and motor dysfunction, may also occur in 30 to 60% of patients that develop clinical disease [9,10,11]. Although currently regarded as a top priority zoonotic disease for the US population [12], there are no standard treatment guidelines outside of supportive care, nor is there an FDA-approved drug or vaccine available for the treatment or prevention of WNV neuroinvasive disease, respectively [8].

WNV pathogenesis is characterized by three phases: (1) an early phase of skin infection and spread to the local draining lymph nodes following a bite from an infected mosquito, (2) viral dissemination to peripheral organs, and (3) invasion of the central nervous system (CNS) [13]. To fight the invasion of WNV, the mammalian host mobilizes three lines of defense: the skin and the innate immunity at the early phase followed by the adaptative (humoral and cellular) immunity at later stages [13,14].

**Figure 1:**
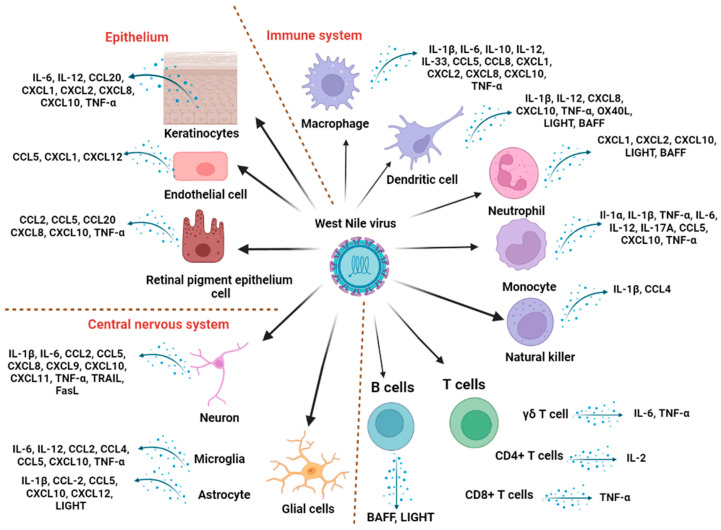
Cellular targets of West Nile virus and corresponding cytokine response in mammals. The illustration was created in Biorender.com. Abbreviations: BAFF: B-cell activating factor; FasL: Fas ligand; TNF-α: tumor necrosis factor -α, TRAIL: TNF-related apoptosis-inducing ligand.

Cytokines are signaling proteins that are expressed by many immune and nonimmune mammalian cells (Figure 1). Their induction and regulation are tightly linked to WNV replication during the early phase of infection [15,16,17,18,19,20]. While they are engaged in all three lines of defense against WNV, they also contribute to immune-mediated tissue damage in the brain. Among these cytokines, interleukins (ILs), chemokines, and tumor necrosis factor superfamily (TNFSF) ligands are major players in immunity against WNV, as evidenced by transcriptome profiling of WNV-infected cells and tissues using DNA microarrays or RNA sequencing [21]. Several reviews have shed light on their role in flaviviral infections in general [22,23,24] and in specific flaviviral diseases, including dengue [25,26] and Zika [27] viruses. Closely related flaviviruses elicit different immunomodulation profiles in their hosts [28,29,30] and differentially antagonize antiviral pathways [31]; however, WNV pathogenesis appears to have unique aspects compared with other neurotropic viruses [28,32], which will be discussed throughout this review. Therefore, it is important to address the role of cytokines in the specific context of WNV infection.

There is mounting evidence that the host immune response, driven by cytokines, plays a pivotal role in the pathogenesis of WNV and the outcome of the disease. First, clinical data support that diverse cytokine profiles, depending on the sex [33], health condition [34], and human polymorphisms in these immune-coding genes [35,36,37] correlate with different outcomes of the infection, thus inciting the use of related biomarkers to predict the severity of WNV disease in a clinical setting [33]. Secondly, using cytokines as agonists or blocking their effects via pharmaceutical or genetic means in murine models demonstrated their ability to completely alter WNV-associated disease phenotypes [16,38,39,40,41,42,43,44,45,46,47,48,49]. Therefore, an improved understanding of the participation of cytokines in the pathogenesis of WNV may not only help optimize diagnosis and prognosis, but also guide research of immune-modulatory strategies to treat WNV-induced neurological disease.

In this review, we summarize findings from clinical studies as well as experiments conducted over the past two decades using in vitro and in vivo WNV infection models to recapitulate ILs, chemokines, and TNFSF ligands that participate in WNV infection, indicate those of a known relevance in WNV pathogenesis, and identify candidates needing further research to undercover their pertinence as therapeutic targets.

## 2. Interleukins (ILs) in WNV Infection

ILs are proteins that modulate cell growth, differentiation, and activation during antiviral response [50]. WNV induces the release of at least 22 ILs in mammalian hosts (Table S1). To date, IL-1β, IL-6, IL-10, IL-12, IL-17A, IL-22, and IL-23 have been directly investigated (Table 1), while little information is available about the remaining ILs involved in the immune response to WNV infection.

### 2.1. Interleukin-1 Family

Currently, 11 cytokines are considered members of the IL-1 family: IL-1α, IL-1β, the IL-1 receptor antagonist [IL-1ra], IL-18, IL-33, IL-36α, IL-36β, IL-36γ, IL-36ra, IL-37, and IL-38 [113,114]. Among them, IL-1α [19,76,87,115], IL-1β [10,16,51,52,53,80,94,116], IL-1ra [16,29,77], IL-18 [117], and IL-33 [118] are known to be released in response to WNV infection.

IL-1 is an extremely potent inflammatory cytokine, triggered in response to WNV infection both in vitro and in vivo, in the periphery and in the CNS (Table S1). The role of IL-1 in WNV infection has been studied mainly through murine models deficient in IL-1R1 and unable to respond to IL-1α, IL-1β, or IL-1ra [16,54,66,115]. IL-1R1 signaling conferred protection to mice against WNV disease and mortality [16]. During early WNV encephalitis, IL-1R1 controlled viral replication and subsequent apoptosis within neurons [16,115]. Further, IL-1 controlled leukocyte infiltration as well as T cell responses in the CNS [54,66,115] and restrained inflammation by downregulating pro-inflammatory cytokines, such as TNF-α and IL-6 [16] and chemokines, such as CCL2 and CCL5 [16,51,54]. Intracranial injection of WNV in wild-type C57BL/6 mice led to paradoxical results concerning the direct effect of IL-1 on viral replication in the brain. In fact, while IL-1 did not directly impact viral replication within the CNS in some studies [51,53,115], IL-1 was found to mediate CNS-intrinsic virus restriction in another study [16]. The disparities between these studies despite the use of the same model of infection could be in part explained by the differences in the viral strains used to infect the mice.

The pro-inflammatory IL-1α and anti-inflammatory IL-1ra cytokine expression patterns and roles during WNV infection are still not clear. In human sera, IL-1ra expression was variable in WNV-infected presymptomatic and asymptomatic donors [29], but upregulated during acute WNV infection [16,34]. While no human study has yet reported IL-1α regulation during WNV natural infection, IL-1α modulation during WNV infection varies across studies using experimental models (Table S1).

IL-1β is a key player in early, acute, and severe WNV pathogenesis. Indeed, IL-1β is one of the earliest cytokines detected following an infected mosquito bite in murine models [22,62,119]. Further, this cytokine mediated epidermal dendritic cell (DC) and Langerhans cell migration from the epidermis to the local draining lymph nodes [22,62,119]. In mouse brains, IL-1β was secreted during the acute phase, mainly by infiltrating/resident macrophages [54,63] and even later during recovery, mainly by astrocytes [63]. Current evidence demonstrates that IL-1β plays a dual role during WNV-induced disease, being protective during the acute phase, and driving neurological sequela in the long term. Mice deficient in both IL-1β signaling and Apoptosis-associated speck-like protein containing C-terminal caspase recruitment domain (ASC), which induces caspase-1-dependent inflammasome activation and IL-1β production, were found to increase WNV viral titers and disease severity specifically in the CNS [16,51]. Further, in humans with a history of asymptomatic or severe WNV infection, decreased IL-1β induction in their peripheral blood mononuclear cells and macrophages was a hallmark of severe disease [94]. In blood donors who tested positive for WNV RNA after routine blood screening, IL-1β was upregulated in the plasma for six months after their initial blood donation and correlated inversely with WNV RNA loads [16]. In mice, such a sustained IL-1β overexpression, induced specifically by the NOD-like receptor-pyrin-containing proteins 3 (NLRP3) inflammasome cleavage in astrocytes after the recovery period of WNV encephalitis [16,22,63,119], resulted in defective spatial learning and synaptic recovery [53,63]. Thus, improper NLRP3 inflammasome activation and IL-1β secretion in the brain is currently considered as a plausible mechanism for the development of long-term neurological sequela after WNV infection [120].

IL-18 is also a pro-inflammatory cytokine produced following inflammasome activation [121,122]. WNV infection of human primary monocyte-derived DCs or transformed human neuroblastoma cell line (SK-N-SH, ATCC HTB-11™) did not increase IL-18 production [52,117]. However, IL-18 was upregulated in spleen and lung tissues of WNV-infected mice [76]. IL-18 is suggested to further immunopathogenesis of DENV [123], but no investigations have been conducted yet to test this during WNV infection.

Another member of the IL-1 family, IL-33, was upregulated in splenic macrophages of WNV-infected mice [118]. IL-33 signaling through the ST2 receptor can trigger pro-inflammatory and anti-inflammatory responses [124,125]. Generally, in viral infections, IL-33 is considered a protective agent through enhancing CD8+ T cell responses [126] and attenuating viral encephalitis by downregulating iNOS expression in the CNS [127]. Therefore, with this understanding, promoting the activity or production of this cytokine during WNV infection could present therapeutic advantages. More work is needed to investigate the functions of this cytokine in the context of WNV infection.

### 2.2. Interleukin 6 Family

IL-6 is a pleiotropic cytokine involved in many biological processes, including immune responses, hematopoiesis, bone metabolism, and embryonic development [128]. It is one of the most important cytokines during a viral infection [129], and studies using different experimental models describe IL-6 changes during WNV infection (Table S1). Human cytokine studies following WNV infection highly suggest an important role for IL-6. Acute infection in humans could induce a high synthesis of IL-6 in the CSF [130] and the serum [130,131] of patients with WNV fever and WNV neuroinvasive disease. Further, in another study, IL-6 levels in the serum were lower in healthy viremic individuals compared to uninfected individuals before and after IgM seroconversion [124]. IL-6 prolonged expression has been reported in the serum of individuals who experience severe long-term fatigue following symptomatic WNV infection [132]; however, no studies have yet been performed to support the causal relationship between IL-6 levels and WNV-associated disease severity in humans. A single in vivo study investigated the involvement of IL-6 in WNV infection [67] and described that, when infected with WNV, IL-6-deficient mice exhibited similar mortality rates as wild-type mice [67]. Further work is needed to clarify whether this is due to a minor role of this cytokine in WNV infection or to the specific experimental conditions used in this study.

### 2.3. Interleukin 17 Family

Currently, there are 6 inflammatory cytokines that represent the IL-17 family, namely IL-17A, IL-17B, IL-17C, IL-17D, IL-17E, and IL-17F [133]. Among these, IL-17A, a pro-inflammatory cytokine, is upregulated in vitro [43] and in vivo [43,76,85] following WNV infection (Table S1). In humans, in the absence of symptoms, increased IL-17 levels were found in WNV-infected individuals when compared to levels from non-infected blood donors [29]. On the contrary, very low serum levels of IL-17A, as well as complete absence of IL-17A expression in the CSF [130], were found in both febrile and neuroinvasive disease patients. In four serologically confirmed WNV patients with persistent post-infectious symptoms, no increase in IL-17 could be detected [125]. These findings, suggesting a link between IL-17A expression and a favorable outcome of WNV human infection, are supported by one in vivo study in mice, wherein they found that IL-17A facilitated WNV clearance by inducing expression of cytotoxic mediator genes and promoting CD8+ T cell cytotoxicity [43].

### 2.4. Interleukin 12 Family

The IL-12 family includes four members: IL-12, IL-23, IL-27, and IL-35 [134], among which IL-12 and IL-23 are upregulated in vivo following WNV infection (Table S1). IL-12 is composed of two covalently linked subunits, p40 and p35, which form when combined with the bioactive IL-12p70 [134]. IL-23 also comprises two subunits, p19 and p40, and the latter is shared with IL-12 [134]. Currently, no human studies have highlighted IL-23 changes following WNV infection, but IL-12 was reported to be highly expressed in presymptomatic and asymptomatic WNV-infected blood donors [29] and unchanged in symptomatic WNV-infected blood donors during the early phase of infection [34]. Cytokine analyses in WNV-infected individuals confirmed that IL-12p70 could be overexpressed in the serum for months [132] and even years [135] after infection. Mice deficient in individual subunits of IL-12 (p35) or IL-23 (p19) or the shared p40 subunit were used to determine the specific role of each cytokine. Animals deficient in IL-12p40 or IL-23p19, but not IL-12p35, had decreased leukocyte homing to the brains and increased mortality, supporting the importance of IL-23 in protective immune cell infiltration and homing during the acute phase of infection [82]. More research is warranted to cast light on the participation of these cytokines during the recovery phase from WNV infection.

### 2.5. Interleukin 10 Family

The IL-10 family of cytokines contains IL-10, IL-19, IL-20, IL-22, IL-24, IL-26, IL-28, and IL-29 [114], among which IL-10 and IL-22 expression are upregulated in models of WNV infection (Table S1). Increased levels of IL-10 were detected in the plasma of acute, viremic [124], asymptomatic blood donors diagnosed with WNV [29]. However, no significant difference in the IL-10 in serum [130,131] and CSF [130] samples from patients with WNV fever and WNV neuroinvasive disease was found [130]. Genetic or pharmacologic blockade of IL-10 signaling helped to increase survival after WNV lethal challenge in mice [79], and additional studies corroborate a pathogenic role of IL-10 in acute WNV infection. First, previous sensitization to salivary proteins delivered by multiple *A. aegypti* bites resulted in an increased IL-10 expression associated with an aggravated disease [136]. Second, in mice infected with a hamster-derived WNV strain, a decreased IL-10 production correlated with lower frequency of virus persistence in the spleen compared to that of WNV NY99-infected mice [78]. The only study to investigate IL-22 described a minimal effect in the periphery, but mice deficient in IL-22 were more resistant to lethal WNV infection, and IL-22 promoted early entry of virus-carrying neutrophils into the CNS by regulating chemotaxis (mainly via Cxcr2 signaling) at the blood–brain barrier (BBB) [42].

## 3. Chemokines in WNV Infection

Chemokines are chemotactic cytokines that bind to G protein-coupled receptors to direct cell movement during homeostasis and inflammation [114]. These proteins are divided into four subfamilies: C chemokine, CC chemokine, CXC chemokine, and CX3C chemokine, based on the number and positioning of conserved N-terminal cysteine residues [114]. Changes in the expression of chemokines and their receptors have been observed in response to WNV infection in mammalian models (Table S2). Studies focusing on chemokines receptors, including Ccr2, Ccr5, Ccr7, Cxcr2, Cxcr3, Cxcr4 and Cx3cr1 in WNV-infected models have helped define the importance of chemokines in a time- and organ-specific manner (Table 1). However, each of these receptors can be bound to several chemokines, and few reports concerning the participation of chemokines in WNV infection exist to date, including CCL2, CCL7 and CXCL10 (Table 1). Thus, the precise importance of individual chemokines during WNV infection demands additional research.

### 3.1. CC Chemokines

#### 3.1.1. CCL2, CCL7 and CCL12 (Ccr2 Agonists)

Ccr2 and its ligands play important roles in monocytes mobilization under inflammatory conditions [137], and they can be induced following experimental WNV infections (Table S2). Ccr2 agonist CCL2 is highly expressed during human WNV infections. The CCL2 gene expression is upregulated in the brain tissues of patients that succumb to WNV encephalomyelitis [56]. Accordingly, CCL2 production was significantly elevated in the serum of WNV-infected patients [124], with male blood donors having higher levels of CCL2 than the female blood donors in the post-IgM phase [33]. Further, elevation of CCL2 in the post-IgM seroconversion phase was associated with improved symptom outcomes following WNV infection [33,34]. During WNV infection, Ccr2 activation induced monocytosis dependent on CCL2 and CCL7, but not CCL12, and protected mice from lethal challenge mainly by regulating blood monocyte levels [44]. CCL2 mediated monocyte migration to the infected dermis and the draining lymph node, as well as their return from the blood to the bone marrow and their differentiation into DCs during the early phase in WNV-infected mice [89]. CCL2 also mediated accumulation of inflammatory monocytes into the brain, and their differentiation to microglia decreased survival, thus playing a pathogenic role in WNV encephalitis [90]. However, in another study using murine models, CCL2 was only partly involved in monocyte recruitment and did not play a pivotal role in survival following lethal challenge [45]. In contrast, CCL7 deficiency resulted in increased viral burden in the brain, enhanced mortality, and delayed migration of neutrophils and CD8+ T cells into the CNS [45]. CCL7 was significantly decreased in humans with a worse outcome as compared to those with a better outcome in the post-IgM phase [34]. Whereas the role of CCL2 in WNV pathogenesis remains unresolved, CCL7 appears to have favorable effects, improving the outcome of WNV infection.

#### 3.1.2. CCL3, CCL4, and CCL5 (Ccr5 Agonists)

CCR5 and its interaction with chemokine ligands mediate chemotactic activity in leukocytes and are involved in hematopoiesis and immune response [138]. CCL3, 4 and 5 chemokines binding the chemokine receptor Ccr5 could not be detected in human sera during the early and late phases of infection [34,124] but were strongly induced in the CNS of mice after experimental WNV infection (Table S2). In humans, Ccr5 deficiency did not predispose to WNV infection, but once infected, patients could be particularly susceptible to present early and late clinical manifestations if their Ccr5 function was missing or blocked [35,36,139]. In line with these findings, studies in mice describe that Ccr5 deficiency led to increased symptomatic disease and mortality after subcutaneous infection with WNV, although Ccr5 was not required for cell-mediated immunity in the periphery [32,93]. WNV-infected Ccr5-/- mice had a significantly decreased ability to recruit antiviral mononuclear cells specifically into their WNV-infected brain, increased BBB permeability, and elevated levels of Ccr5 ligands [32,93]. The individual roles of the Ccr5 ligands remain unclear, as no in vivo models have been applied to address their contribution in WNV pathogenesis. One in vitro study described that induction of CCL5 in response to WNV infection was not sufficient to promote leukocyte transmigration across the endothelial layer in a model of the BBB containing both endothelial cells and astrocytes [140].

#### 3.1.3. CCL19 and CCL21 (Ccr7 Agonists)

Interactions between Ccr7 and its cognate ligands are involved in the induction of inflammatory and T cell responses [141]. WNV infection in murine models supports that Ccr7 and ligands CCL19 and CCL21 could be upregulated at the gene levels [64,76,110] and contribute to the host resistance against WNV. Chemokine receptor Ccr7 was essential for survival following a WNV lethal challenge in mice [46]. Further, Ccr7 was required for myeloid cells’ infiltration into the lymph nodes and restricted their entry into the brain, assisting in viral clearance and decreasing pathological effects of an excessive cytokine production [46].

### 3.2. CXC Chemokines

#### 3.2.1. CXCL1-3, CXCL6-8 (Cxcr2 Agonists)

Cxcr2 plays a nonredundant role in mediating trafficking of neutrophils, which are suggested as a carrier of WNV in the blood [49]. Mice deficient in Cxcr2 had a similar mortality rate as wild-type mice, although their time to death was delayed [49]. Cxcr2 binds to CXCL1, CXCL2, CXCL3, CXCL5, CXCL6, CXCL7, and CXCL8 [114], which are all modulated by WNV infection (Table S2). CXCL8 is upregulated in WNV-infected primary human cultures [88] and cell lines [18,52,57,75,88,98] as well as brain and spinal cord samples from experimentally infected Rhesus monkeys (*Macaca mulatta*) [77]. Genes associated with CXCL8 production and upregulation were induced in New Zealand white rabbits (*Oryctolagus cuniculus*) [68]. CXCL8 is also detected at high levels in WNV-infected individuals [131,135]. Further, patients with more severe symptoms during the early phase of infection had significantly higher CXCL8 expression in their serum when compared to WNV-negative controls [34]. These findings suggest an important role of this cytokine in the pathogenesis of natural infections in humans. Yet, to date, no in vivo studies investigate this observation. This can be explained by the lack of true CXCL8 homologs in mice [142], which are currently the most widely used animal models to study WNV pathogenesis. Using alternative WNV models of infection that have an ortholog CXCL8 gene, such as rabbits [70,143,144,145,146] and nonhuman primates, [77,147] will be necessary to circumvent this issue.

#### 3.2.2. CXCL9 and CXCL10 (Cxcr3 Ligands)

Cxcr3 and its ligands are responsible for T-cell trafficking, activation, differentiation, and functions [48]. WNV natural infection in humans can induce high levels of CXCL9 [124] and CXCL10 [34,124,131,135] in the serum. Likewise, these chemokines were elevated following WNV infection in various experimental models (Table S2). Evidence from these models suggests that Cxcr3 signaling can have multifaced roles during WNV infection. In vitro, downregulation of neuronal CXCR3 signaling through TNF receptor 1 (TNFR1) decreased CXCL10 and resultant apoptosis following WNV infection [99]. In vivo, Cxcr3 had no effect in WNV replication or clearance in peripheral lymphoid tissues [47]. However, CXCL10, but not CXCL9, and its cognate receptor Cxcr3 were required for survival after lethal WNV challenge and regulated the CD8+ T cells migration and clearing of WNV infection in the brain compared to control mice [47,48]. This can explain the evidence of both protective and deleterious effects of CXCL10 in humans. In fact, higher susceptibility to WNV in blood donors was marked by lower levels of CXCL10/IP-10 during the post-IgM phase [33,34]. Importantly, analysis of autopsied neural tissues from humans with WNV encephalomyelitis revealed upregulation of CXCL10-coding gene [56] and symptom development was positively correlated with CXCL10/IP-10 production during the earliest phase of disease [34]. In later stages, significantly higher serum levels of CXCL10 were detected in patients with prolonged post-infection fatigue (>6 months) after symptomatic WNV infection [132]. Thus, CXCL10 transition from driving protective immune responses to deleterious ones needs further research as a possible therapeutic target.

#### 3.2.3. CXCL12 (Cxcr4 Ligand)

Cxcr4 is the most widely expressed chemokine receptor and is involved in cell migration, hematopoiesis, and cell homing [148]. Changes in the expression of Cxcr4 and its canonical ligands CXCL12 can be induced following experimental WNV infection (Table S2), whereas their expression patterns in WNV-infected patients is still unclear. Current evidence from experimental infections suggests that CXCL12 favors WNV neuropathogenesis. In fact, CXCL12 expression, which was mediated by IL-1 at the CNS microvasculature [54], restricted entry of T cells at the BBB and prevented virus-specific CD8+ T cells from clearing WNV within the CNS parenchyma, resulting in enhanced mortality in a murine model of infection [100].

### 3.3. CX3C Chemokines

Chemokine CX3CL1 and its receptor CX3CR1 can exert pro-inflammatory or anti-inflammatory responses [149]. Their coding genes were upregulated in vivo following WNV infection in B6129PF2 and C57BL6/J mice [32] and Rhesus monkeys [77] (Table S2). Investigation in a murine model did not support their having a role that aids in survival against WNV infection [32].

## 4. Tumor Necrosis Factor Superfamily Ligands

Interactions between TNFSF ligands and their cognate receptors control the survival, proliferation, differentiation, and functions of immune cells [111]. Among the TNFSF ligands, TNF-α [72,99,103,104,105], Fas ligand (FasL) [39,76,110], TNF-related apoptosis-inducing ligand (TRAIL) [69,109,110], CD40L [85,111], B-cell activating factor (BAFF) [112], TNF-related weak inducer of apoptosis (TWEAK) [85], OX40L [117], and tumor necrosis factor superfamily member 14 (LIGHT) [76,150] are implicated in WNV pathogenesis (Table S3).

### 4.1. Tumor Necrosis Factor α

TNF-α, a cytokine having pro- and anti-inflammatory properties [151], has inconsistent expression patterns following WNV infection in humans. Human sera analysis during WNV fever and WNV neuroinvasive disease showed no detectable change in TNF-α expression [131], but others describe a significant TNF-α upregulation in WNV-infected patients during the acute phase [124,130] and even long after the virus had presumably been cleared by the immune system [125]. TNF-α was significantly higher in individuals with a history of WNV infection and subsequent development of chronic kidney disease compared to healthy individuals [135]. In accordance with the latter human reports, almost all studies using experimental models describe increased TNF-α during WNV infection (Table S3). Studies investigating the importance of TNF-α in the pathogenesis of WNV infection indicate that this cytokine has a limited role in controlling WNV infection in peripheral organs [38,104], with no consensus regarding the signaling cascade and contribution to control WNV in the CNS (Table S3). For instance, TNF-α receptor 1 (TNF-R1) signaling was suggested downstream of Toll-like receptor (TLR)-3, as TLR3 deficiency led to impaired TNF-α production during WNV infection in microglia [67], but the same observation did not occur in bone marrow-derived DCs [152]. Whereas in one study, mice deficient in TNF-R1 had a mortality rate significantly greater than that in wild-type mice after WNV challenge [104], the opposite phenomenon was observed in another study using the same model [67]. The former study suggested that TNF-α interaction with TNF-R1 protected mice against WNV infection by regulating migration of inflammatory cells into the brain during acute infection [104], while the latter suggested that TNFα could be responsible for early WNV neuroinvasion due to increased permeability of the BBB [67]. Immunization of mice with salivary gland components led to early production of TNF-α following WNV infection, which aligned with a delay in CNS infection and significantly lower WNV brain titers compared to mock-immunized mice [153], suggesting a protective role during WNV encephalitis. However, another study described higher TNF-α levels that corroborated an increased pathogenicity of neuroinvasive WNV variants compared to non-neuroinvasive variants in mice, [73] and TNF-α was involved in WNV-induced neurotoxicity [52]. Additional research is necessary.

### 4.2. TRAIL and FasL

TRAIL and FasL activate apoptosis through cell surface death receptors [111]. These cytokines are upregulated on the gene levels using in vivo models, including mice (Table S3). In mouse models, TRAIL contributes to disease resolution [38], while the role of FasL remains elusive [28,39]. In mice, TRAIL genetic deficiency increased susceptibility to lethal WNV challenge, and CD8+ T cells encountered difficulty in clearing WNV from neurons [38]. WNV induced expression of Fas in neurons, functional FasL was required to protect IFNγ-deficient C57BL/6 mice from lethal WNV infection, and CD8+ T cells utilized FasL to restrict WNV infection in neurons [39]. However, another study using the same mice deficient in either Fas or FasL did not find differences in mortality or viral burden in the brain [28]. Inconsistent results from these studies could be attributed to the differences in the viral strains (WNV 3000.0259 strain [39] versus WNV Sarafend strain [28]) as well as the route of animal infection (footpad [39] versus intravenous route [28]).

### 4.3. CD40L

CD40L is a modulator of a wide range of humoral and cellular immune responses [111] and is regulated by WNV infection in the mouse brain [64]. In mice, CD40-CD40L interactions were required for protection from lethal WNV challenge, efficient antibody production by B cells, and T-cell migration across the BBB [40]. While there is evidence to suggest a role for CD40L in WNV infection, more research is needed.

### 4.4. BAFF

BAFF is required for peripheral B-cell survival and homeostasis and is upregulated in mice neutrophils and DCs after WNV challenge [112]. BAFF signaling was essential for survival against lethal WNV infection in mice [41]. BAFF from DCs, not neutrophils, helped to sustain or promote B-cell humoral responses to WNV, since WNV-specific antibody responses were decreased in mice lacking BAFF expression on DCs [112]. Further, BAFF receptor-deficient mice were susceptible to WNV infection but could develop sustained protective immunity when treated with immune sera from a wild-type mouse with antibodies to WNV [41].

## 5. Conclusions

Cytokine characterization represents a major advance in our understanding of the overall regulation of WNV-driven immune responses. Cytokine signaling of IL-1β, IL-23, IL-17A, CCL7, CXCL10, TRAIL, CD40L, and BAFF provides protection against acute WNV infection in mice; IL-10 and IL-22 aid in WNV pathogenicity; IL-6 and IL-12 had no apparent effect during infection; and CCL2, TNF-α, and FasL roles remain elusive. Determining the exact function of a particular cytokine can be challenging and underscores the most important messages from this review: First, the biological context, such as the cellular source, the target, the phase of the immune response, and the presence or absence of other cytokines influences their expression pattern and function. Experimental conditions, varying across the studies, such as viral strains or passages, laboratory investigation techniques, and time-points of sample harvesting, might also explain inconsistent, sometimes paradoxical results regarding the roles of cytokines during WNV infection. Second, the outcomes of WNV infection depend not only on viral clearance but also on the extent of the inflammatory response driven by cytokines. In fact, WNV infections in humans and laboratory animals provide evidence that pro-inflammatory cytokines, such as IL-1β, TNF-α, IL-12p70, CXCL10, and IL-6, can be chronically elevated after WNV is cleared. This indicates that an effective treatment against WNV neuroinvasive disease should include anti-inflammatory drugs to treat the exacerbated inflammatory response during the acute phase and to prevent long-term neurological sequalae, as these cytokines are linked to neuronal injury in several neurodegenerative diseases [154]. Future studies are critical to understanding how regulation of these cytokines can improve the course of illness. This can be accomplished by studying existing drugs or small molecules against the aforementioned cytokines, as well as development of new therapeutics that interfere with these cytokine pathways. Finally, this review highlights the need for additional research into these cytokines, considering the biological importance they maintain, which will help identify immunomodulatory therapeutic targets against WNV neuroinvasive disease. For example, alternative infection models should be developed for the study of CXCL8, hampered to date by the lack of true homologs in rats and mice. More work aimed at dissecting the roles of important cytokines depicted from clinical human studies, such as IL-15, CCL8, CCL11, CCL13, and CCL20 is warranted to understand their contribution to the immunopathogenesis of WNV infection.

## Figures and Tables

**Table 1:** Summary of interleukins, chemokines, chemokine receptors and tumor necrosis factor ligands triggered following West Nile virus (WNV) infection, whose pathogenesis has been studied in mice models in vivo.

	Survival Following Lethal WNV Challenge ^1^	Role in WNV Pathogenesis ^1^	References
**Interleukins**			
**IL-1 β**	+	Langerhans cell migration to the draining LNs. Protective CNS-intrinsic immune response and leukocytes migration to the CNS. Synaptic deficits and spatial learning defects during recovery.	[16,18,32,51,52,53,54,55,56,57,58,59,60,61,62,63,64,65]
**IL-6**	N.E.	N/A	[19,20,52,65,66,67,68,69,70,71,72,73,74,75]
**IL-10**	−	Viral replication in the periphery and in the CNS and downregulation of IL-12/23 p40 and TNF-α in the CNS.	[19,32,61,64,68,73,76,77,78,79]
**IL-12**	N.E.	No role of IL-12 p35 in brain infiltration or homing of leukocytes.	[66,70,76,80,81,82,83,84]
**IL-17 A**	+	CD8+T cell cytotoxicity.	[43,76,85]
**IL-22**	−	WNV entry into the CNS via neutrophils Intrinsic control of viral replication in the brain CXCL1, CXCL5 and Cxcr2 expression in the brain	[42,61,76,86]
**IL-23**	+	Brain infiltration and homing of leukocytes	[76,82]
**CC chemokines**			
**CCL2**	+/−	Monocytes migration and differentiation into DCs in the skin and LNs. Monocytosis and monocytes trafficking to the brain.	[19,20,32,44,45,47,56,64,65,69,76,77,84,85,87,88,89,90,91,92]
**CCL7**	+	Monocytosis, recruitment of neutrophils and CD8+ T cells into the CNS Viral clearance from the brain.	[44,45,47,76,85,91]
**C chemokine receptors**			
**Ccr2**	+	Monocytosis, monocytes migration to the brain and viral clearance from the CNS.	[44,76]
**Ccr5**	+	Leukocyte trafficking to the CNS/control of the BBB permeabilityViral clearance in the brain	[32,93]
**Ccr7**	+	DCs and T cell trafficking to the LNs Control of WNV-infected myeloid cells infiltration into the CNS.	[46]
**CX chemokine**			
**CXCL10**	+	Recruitment of CD8+ T-cells into the CNS.	[19,20,32,47,48,49,56,57,64,65,68,70,71,75,76,77,80,84,85,87,88,91,92,94,95,96,97,98]
**CX chemokine receptors**			
**Cxcr2**	−	N/A	[49]
**Cxcr3**	+	CD8+ T cells control of WNV infection within the cerebellum	[48,99]
**Cxcr4**	−	Downregulation of T cells trafficking to the brain	[100]
**CX3C Chemokine**			
**CX3CL1**	N.E.	Monocytes (microglial precursor) recruitment to the brain.	[32]
**CX3C Chemokine receptor**			
**Cx3cr1**	N.E.	N/A	[32,46,90]
**Tumor necrosis factor superfamily ligands**			
**TNF-α**	+/−	No effect in Langerhans cell migration to the draining lymph nodes. Regulation of leukocyte infiltration in the CNS. Down-regulation of neuronal Cxcr3 and subsequent neuronal apoptosis.	[17,19,20,32,52,55,57,61,62,64,65,66,67,68,69,70,72,73,74,75,76,77,84,87,88,95,99,101,102,103,104,105,106,107,108]
**TRAIL**	+	CD8+ T cells-mediated viral clearance in the CNS.	[38,69,109]
**FasL**	+/N.E.	CD8+ T cells-mediated viral clearance in the CNS.	[39,59,76,77,110]
**CD40L**	+	Efficient production of neutralizing antibodies, trafficking of CD8+ T cells into the brain, and control of WNV replication in the CNS.	[111]
**BAFF**	+	Viral clearance from sera, spleen, and brain.	[41,112]

**^1^** Data from experiments using mice (*Mus musculus)* in vivo as WNV-infection models. (+): Enhances survival rates; N.E: No effect (no difference between cytokine-deficient and control groups); (−): Enhances mortality rates; N/A: Not available. Abbreviations: BAFF: B-cell activating factor; BBB: Blood–brain barrier; CNS: Central nervous system; LNs: Lymph nodes; TNF-α: Tumor necrosis factor -α, TRAIL: TNF-related apoptosis-inducing ligand.

## Data Availability

Not applicable.

## Supplementary Materials

The following supporting information can be downloaded at: https://www.mdpi.com/article/10.3390/v15030806/s1, Table S1: Interleukins involved in West Nile virus pathogenesis; Table S2: Chemokines involved in West Nile virus pathogenesis; Table S3: Tumor necrosis factor superfamily ligands involved in West Nile virus pathogenesis.
