# Supplementary material for: Interleukins, Chemokines, and Tumor Necrosis Factor Superfamily Ligands in the Pathogenesis of West Nile Virus Infection
Source: Viruses. 2023 Mar 22;15(3):806. doi: 10.3390/v15030806 (PMC10053297; doi:10.3390/v15030806)
Supplement: Supplementary file 1 [file viruses-15-00806-s001.zip › viruses-2284274-supplementary.pdf]

<u>Supplementary table S1</u>: Interleukins involved in West Nile virus pathogenesis.

Cytokine	Model	Host	Expression	Source	Role in WNV pathogenesis	Reference
**IL-1 α**	*In vitro*	Horse	Upregulation	PBMCs	Unknown	[1]
	In vivo	Mouse	No change	Blood/Brain		[2–4]
			Upregulation	Blood/brain/ lung/ liver		[5–8]
			Strain-dependent	Brain		[9]
**IL-1 β**	*In vitro*	Human	No change	Astrocytes/ Resting MΦ/ U-937/ BMDCs	Unknown	[10–14]
			Upregulation	Neural stem cells/ U373/ LAN-2/MΦ/ SK-N-SH cells/ Activated MΦ/ NK cells	Direct neurotoxic effects in SK-N-SH cells	[11,12,15–18]
			Strain-dependent	THP-1/ THP-1-derived MΦ	Unknown	[19]
		Pig (*Sus domesticus*)	No change	BMDCs	Unknown	[13]
		Mouse	Upregulation	Primary BMECs and astrocytes	Mediates the ability of CXCR4 expressing lymphocytes to adhere to BMECs via CXCL12 expression.	[20]
				Peritoneal exudate cells	Expression may be induced by WNV envelope protein	[21]
				MΦ and BMDCs	Reduces WNV replication. Protective via the modulation of type I IFN responses dependent on the IRF3.	[22]
				BMDCs	Induced by WNV via ASC	[23]
				Cortical neurons	Reduces WNV replication. Synergizes with type I IFN to induce ISGs.	[2]
			Strain-dependent	MΦ	Unknown	[24]
	In vitro/ in vivo	Horse	Upregulation	PBMCs	Unknown	[1]
	In vivo	Horse	Upregulation	Lymphoid tissue	Unknown	[25]

		Mouse	-	-	Mediates langerhans cell migration and accumulation in the draining lymph nodes upon WNV cutaneous infection	[26]
			Variable	Popliteal lymph node/spleen	Unknown	[2,27]
			No change	Serum		
			Upregulation	Brain	Reduces WNV replication in the brain. Promotes CNS-Intrinsic Immune Control. Prevents synapse recovery and promotes spatial learning defects during WNV recovery	[2–4,27–29]
			Strain-dependent	Brain	Unknown	[9]
		**Rabbit (*Oryctolagus cuniculus*)**	Upregulation	Draining lymph nodes	Unknown	[30]
**IL-1ra**	*In vivo*	Mouse	Upregulation	Spleen/ Lung/ kidney	Unknown	[7]
		NHP (*Macaca mulatta)*	Upregulation	Brain/spinal cord	Unknown	[31]
**IL-2**	*In vivo*	Mouse	Upregulation	Brain	Unknown	[28,32]
	In vitro	Mouse	Upregulation	CD4+ T	Unknown	[33]
**IL-3**	*In vivo*	Mouse	Upregulation	Liver/kidney	Unknown	[7]
			Downregulation	Brain	Unknown	[28]
**IL-4**	*In vitro*	Human	-	BMDCs	No effect on viral replication	[34]
			No change	BMDCs	Unknown	[35]
			Downregulation	BMDCs	Unknown	[36]
	In vivo	Mouse	No change	Brain	Unknown	[27]
			Downregulation	Brain	Unknown	[28]
		Rabbit (*Oryctolagus cuniculus* and *Sylvilagus sp)*	Upregulation	Lymph nodes	Unknown	[37]
**IL-5**	*In vivo*	Mouse	No change	Brain	Unknown	[27]
			Upregulation	Brain	Unknown	[8]
			Downregulation	Brain	Unknown	[28]

IL-6	*In vitro*	Human	No change	Astrocytes/ BMDCs	Unknown	[10,35]
			Upregulation	Microglia/ SK-N-SH/ keratinocytes	Does not interfere with WNV-induced cytotoxicity in SK-N-SH.	[10,16,38]
		Human	Strain-dependent	THP-1/ THP-1-derived MΦ	Unknown	[19]
		Mouse	Upregulation	MΦ/H36.12j/ Renca/ Peritoneal exudate cells/ γδ T cells	Unknown	[21,24,39,40]
			-	N2a/ cortical neurons	Directly responsible for neuronal apoptosis.	[41]
	In vitro/ in vivo	Rabbit	Upregulation	PBMCs	Unknown	[42]
	In vivo	Mice	Upregulation	Blood/ brain	Does not impact mortality rates following lethal WNV-infection/ Production in plasma and brain mediated by Toll-like receptor 3.	[8,29,41,43]
		Rabbit (*Oryctolagus cuniculus* and *Sylvilagus sp)*	Upregulation	Brain/ spleen	Unknown	[37]
	Ex vivo	Mouse	Upregulation	Spinal cord slice	Unknown	[44]
IL-7	*In vivo*	Mouse	Upregulation	Brain	Unknown	[8]
			Strain-dependent	Brain	Unknown	[9]
IL-10	*In vitro*	Human	No change	BMDCs	Unknown	[14,34]
		Mouse	Upregulation	Peritoneal exudate cells/ MΦ	Negatively regulates TNF-α, IL-12/23p40 and type I interferon expression in MΦ.	[21,45]
			-	Splenocytes	Negatively regulates IL-12/23p40 and type I interferon expression in splenocytes.	[45]
	In vivo	Mouse	-	-	Promotes viral replication in the periphery and in the CNS via the I.P. and footpad route, not I.C. route. Decreases IL-12/23 p40 and TNF-α and increases mortality rates	[45]

			Upregulation	Serum/spleen/ lung/ liver/ brain	Unknown	[7,8,27,28,46]
		Rabbit (*Oryctolagus cuniculus* and *Sylvilagus sp)*	Upregulation	Brain/ spleen/ lymph nodes	Unknown	[37]
		Horse	Upregulation	Brain	Unknown	[25]
		NHP (*Macaca mulatta)*	Upregulation	Brain/spinal cord	Unknown	[31]
**IL-11**	*In vivo*	Mouse	Upregulation	Brain	Unknown	[27]
**IL-12**	*In vitro*	Human	No change	BMDCs	Unknown	[14,34,35]
			Strain-dependent	THP-1/ THP-1-derived MΦ	Unknown	[19]
		Mouse	Upregulation	Keratinocytes/ microglia	Unknown	[47,48]
		Rabbit (*Oryctolagus cuniculus)*	Upregulation	PBMCs	Unknown	[42]
	In vivo	Mouse	-	-	IL-12 p35 does not impact survival rate following WNV-challenge/ Does not interfere with brain infiltration or homing of leukocytes.	[49]
			Upregulation	Blood/ Spleen/ lung/ liver/ brain/DCs	Produced via TLR7-MyD88 signaling during WNV infection.	[7,43,50]
**IL-13**	*In vivo*	Mouse	No change	Brain	Unknown	[27]
			Upregulation	Serum/lung/kidney	Unknown	[7,8]
			Downregulation	Brain	Unknown	[28]
			Strain-dependent	Brain	Unknown	[8]
**IL-15**	*In vitro*	Human	No change	BMDCs	Unknown	[35]
	In vivo	Mouse	Upregulation	Spleen/ lung/ brain	Unknown	[7,27,32]
		Horse	Upregulation	Thalamus/ cerebrum	Unknown	[51]
**IL-16**	*In vivo*	Mouse	Downregulation	Brain	Unknown	[28]
**IL-17A**	*In vitro*	Human	Upregulation	PBMCs	Unknown	[52]
	In vivo	Mouse	Upregulation	Serum/ liver/ brain	Protects mice from lethal WNV infection and facilitates CD8+T cell cytotoxicity. IL-17A production during WNV infection in mice largely	[7,32,52]

					depends on IL-23 signaling.	
			Strain-dependent	Brain	Unknown	[8]
**IL-17B**	*In vivo*	Mouse	Downregulation	Brain	Unknown	[28]
**IL-18**	*In vitro*	Human	No change	BMDCs/ SK-N-SH	Unknown	[14,16]
	In vivo	Mouse	Upregulation	Lung/ spleen	Unknown	[7]
**IL-20**	*In vivo*	Mouse	Downregulation	Brain	Unknown	[28]
**IL-22**	*In vivo*	Mouse	-	-	Enhances footpad WNV-lethal infection/ Facilitates WNV Entry into the CNS/ Promotes CXCL1, CXCL5 and Cxcr2 expression	[53]
			Upregulation	Lung/kidney	Unknown	[7]
		Horse	Upregulation	Peripheral blood leukocytes/ Thalamus/ cerebrum/ lymphoid tissue	Unknown	[25,51]
**IL-23**	*In vitro*	Human	No change	BMDCs	Unknown	[14]
	In vivo	Mouse	-	-	Protects mice from lethal WNV-challenge. Mediates brain infiltration and homing of leukocytes.	[49]
			Upregulation	Lung/kidney	Unknown	[7]
**IL-33**	*In vivo*	Mouse	Upregulation	Spleen MΦ	Unknown	[54]

Cell lines: H36.12j; *Mus musculus* (macrophage cell line) ATCC CRL-2449™; Renca *Mus musculus* (Kidney epithelial cells line) ATCC CRL-2947™; retinal pigment epithelium cell line (ARPE-19): L929 ATCC CCL-1™; LAN-2: *Mus musculus* neuroblastoma cell line (RRID:CVCL_1829); U-937: *Homo sapiens* Lymphoma cell line CRL-1593.2™; U373: *Homo sapiens* glioblastoma astrocytoma cell-line; SK-N-SH *Homo sapiens* neuroblastoma cell line ATCC HTB-11™; THP-1: Monocytic leukemia cells ATCC TIB-202 ; N2a : *Mus musculus* (neuroblastoma cell line) ATCC CCL-131™.

Abbreviations: ASC: Apoptosis-associated speck-like protein containing C-terminal caspase recruitment domain [CARD]; BMDCs: Bone marrow-derived dendritic cells; BMECs: Brain microvasculature endothelial cells; BMECs: Brain microvascular endothelial cells; CNS: Central nervous system; DCs: Dendritic cells; RPE: retinal pigment epithelium (RPE), ISGs: Interferon-stimulated genes; I.C. : Intracranial ; I.P.: Intra-peritoneal; IFN: Interferon, IRF: IFN regulatory factor 3; MΦ: Macrophages; NHP: Non-Human primate; PBMCs: Peripheral blood mononuclear cells.

References:

1. Uddin, M.J.; Suen, W.W.; Bosco-Lauth, A.; Hartwig, A.E.; Hall, R.A.; Bowen, R.A.; Bielefeldt-Ohmann, H. Kinetics of the West Nile Virus Induced Transcripts of Selected Cytokines and Toll-like Receptors in Equine Peripheral Blood Mononuclear Cells. *Veterinary Research* **2016**, *47*, 61, doi:10.1186/s13567-016-0347-8.

2. Ramos, H.J.; Lanteri, M.C.; Blahnik, G.; Negash, A.; Suthar, M.S.; Brassil, M.M.; Sodhi, K.; Treuting, P.M.; Busch, M.P.; Norris, P.J.; et al. IL-1β Signaling Promotes CNS-Intrinsic Immune Control of West Nile Virus Infection. *PLoS Pathogens* **2012**, *8*, e1003039, doi:10.1371/journal.ppat.1003039.

3. Garber, C.; Vasek, M.J.; Vollmer, L.L.; Sun, T.; Jiang, X.; Klein, R.S. Astrocytes Decrease Adult Neurogenesis during Virus-Induced Memory Dysfunction via Interleukin-1. *Nat Immunol* **2018**, *19*, 151–161, doi:10.1038/s41590-017-0021-y.

4. Soung, A.L.; Davé, V.A.; Garber, C.; Tycksen, E.D.; Vollmer, L.L.; Klein, R.S. IL-1 Reprogramming of Adult Neural Stem Cells Limits Neurocognitive Recovery after Viral Encephalitis by Maintaining a Proinflammatory State. *Brain, Behavior, and Immunity* **2022**, *99*, 383–396, doi:10.1016/j.bbi.2021.10.010.

5. Durrant, D.M.; Robinette, M.L.; Klein, R.S. IL-1R1 Is Required for Dendritic Cell-Mediated T Cell Reactivation within the CNS during West Nile Virus Encephalitis. *Journal of Experimental Medicine* **2013**, *210*, 503–516, doi:10.1084/jem.20121897.

6. Garcia-tapia, D.; Hassett, D.E.; Mitchell Jr, W.J.; Johnson, G.C.; Kleiboeker, S.B. West Nile Virus Encephalitis : Sequential Histopathological and Immunological Events in a Murine Model of Infection. *Journal of NeuroVirology* **2007**, *13*, 130–138, doi:10.1080/13550280601187185.

7. Peña, J.; Plante, J.A.; Carillo, A.C.; Roberts, K.K.; Smith, J.K.; Juelich, T.L.; Beasley, D.W.C.; Freiberg, A.N.; Labute, M.X.; Naraghi-Arani, P. Multiplexed Digital MRNA Profiling of the Inflammatory Response in the West Nile Swiss Webster Mouse Model. *PLoS Neglected Tropical Diseases* **2014**, *8*, e3216, doi:10.1371/journal.pntd.0003216.

8. Kumar, M.; Roe, K.; O'Connell, M.; Nerurkar, V.R. Induction of Virus-Specific Effector Immune Cell Response Limits Virus Replication and Severe Disease in Mice Infected with Non-Lethal West Nile Virus Eg101 Strain. *Journal of Neuroinflammation* **2015**, *12*, 178, doi:10.1186/s12974-015-0400-y.

9. Kumar, M.; Belcaid, M.; Nerurkar, V.R. Identification of Host Genes Leading to West Nile Virus Encephalitis in Mice Brain Using RNA-Seq Analysis. *Scientific Reports* **2016**, *6*, 26350, doi:10.1038/srep26350.

10. Cheeran, M.C.J.; Hu, S.; Sheng, W.S.; Rashid, A.; Peterson, P.K.; Lokensgard, J.R. Differential Responses of Human Brain Cells to West Nile Virus Infection. *Journal of NeuroVirology* **2005**, *11*, 512–524, doi:10.1080/13550280500384982.

11. Kong, K.; Wang, X.; Anderson, J.; Fikrig, E.; RR Montgomery West Nile Virus Attenuates Activation of Primary Human Macrophages. *Viral Immunol* **2008**, *21*, 78–82, doi:10.1089/vim.2007.0072.

12. Marle, G. Van; Antony, J.; Ostermann, H.; Dunham, C.; Hunt, T.; Halliday, W.; Maingat, F.; Urbanowski, M.D.; Hobman, T.; Peeling, J.; et al. West Nile Virus-Induced Neuroinflammation : Glial Infection and Capsid Protein-Mediated Neurovirulence. *J Virol* **2007**, *81*, 10933–10949, doi:10.1128/JVI.02422-06.

13. García-Nicolás, O.; Lewandowska, M.; Ricklin, M.E.; Summerfield, A. Monocyte-Derived Dendritic Cells as Model to Evaluate Species Tropism of Mosquito-Borne Flaviviruses. *Frontiers in Cellular and Infection Microbiology* **2019**, *9*, 5, doi:10.3389/fcimb.2019.00163.

14. Kovats, S.; Turner, S.; Simmons, A.; Powe, T.; Chakravarty, E.; Alberola-Ila, J. West Nile Virus-Infected Human Dendritic Cells Fail to Fully Activate Invariant Natural Killer T Cells. *Clinical and Experimental Immunology* **2016**, *186*, 214–226, doi:10.1111/cei.12850.

15. Riccetti, S.; Sinigaglia, A.; Desole, G.; Nowotny, N.; Trevisan, M.; Barzon, L. Modelling West Nile Virus and Usutu Virus Pathogenicity in Human Neural Stem Cells. *Viruses* **2020**, *12*, 882, doi:10.3390/v12080882.

16. Kumar, M.; Verma, S.; Nerurkar, V.R. Pro-Inflammatory Cytokines Derived from West Nile Virus (WNV)-Infected SK-N-SH Cells Mediate Neuroinflammatory Markers and Neuronal Death. *Journal of Neuroinflammation* **2010**, *7*, 73, doi:10.1186/1742-2094-7-73.

17. Qian, F.; Chung, L.; Zheng, W.; Bruno, V.; Alexander, R.P.; Wang, Z.; Wang, X.; Kurscheid, S.; Zhao, H.; Fikrig, E.; et al. Identification of Genes Critical for Resistance to Infection by West Nile Virus Using RNA-Seq Analysis. *Viruses* **2013**, *5*, 1664–1681, doi:10.3390/v5071664.

18. Yao, Y.; Strauss-albee, D.M.; Zhou, J.Q.; Malawista, A.; Garcia, M.N.; Murray, K.O.; Blish, C.A.; Montgomery, R.R. The Natural Killer Cell Response to West Nile Virus in Young and Old Individuals with or without a Prior History of Infection. *PLoS ONE* **2017**, *12*, e0172625, doi:10.1371/journal.pone.0172625.

19. Xie, G.; Luo, H.; Tian, B.; Mann, B.; Bao, X.; Mcbride, J.; Tesh, R.; Barrett, A.D.; Wang, T. A West Nile Virus NS4B-P38G Mutant Strain Induces Cell Intrinsic Innate Cytokine Responses in Human Monocytic and Macrophage Cells. *Vaccine* **2015**, *33*, 869–878, doi:10.1016/j.vaccine.2014.12.056.A.

20. Durrant, D.M.; Daniels, B.P.; Klein, R.S. IL-1R1 Signaling Regulates CXCL12-Mediated T Cell Localization and Fate within the CNS during West Nile Virus Encephalitis. *J Immunol* **2014**, *193*, 4095–4106, doi:10.4049/jimmunol.1401192.

21. Sapkal, G.N.; Harini, S.; Ayachit, V.M.; Fulmali, P. V; Mahamuni, S.A.; Bondre, V.P.; Gore, M.M. Neutralization Escape Variant of West Nile Virus Associated with Altered Peripheral Pathogenicity and Differential Cytokine Profile. *Virus Research* **2011**, *158*, 130–139, doi:10.1016/j.virusres.2011.03.023.

22. Aarreberg, L.D.; Wilkins, C.; Ramos, H.J.; Green, R.; Davis, M.A.; Chow, K.; Gale, M. Interleukin-1 β Signaling in Dendritic Cells Induces Antiviral Interferon Responses. *mBio* **2018**, *9*, e00342-18, doi:10.1128/mBio .00342-18.

23. Kumar, M.; Roe, K.; Orillo, B.; Muruve, D.A.; Nerurkar, V.R.; Gale, M.; Verma, S. Inflammasome Adaptor Protein Apoptosis-Associated Speck-Like Protein Containing CARD (ASC) Is Critical for the Immune Response and Survival in West Nile Virus Encephalitis. *Journal of Virology* **2013**, *87*, 3655–3667, doi:10.1128/jvi.02667-12.

24. Shirato, K.; Miyoshi, H.; Kariwa, H.; Takashima, I. The Kinetics of Proinflammatory Cytokines in Murine Peritoneal Macrophages Infected with Envelope Protein-Glycosylated or Non-Glycosylated West Nile Virus. *Virus Research* **2006**, *121*, 11–16, doi:10.1016/j.virusres.2006.03.010.

25. Bielefeldt-ohmann, H.; Bosco-lauth, A.; Hartwig, A.; Uddin, M.J.; Barcelon, J.; Suen, W.W.; Wang, W.; Hall, R.A.; Bowen, R.A. Microbial Pathogenesis Characterization of Non-Lethal West Nile Virus (WNV) Infection in Horses : Subclinical Pathology and Innate Immune Response. *Microbial Pathogenesis* **2017**, *103*, 71–79, doi:10.1016/j.micpath.2016.12.018.

26. Byrne, S.N.; Halliday, G.M.; Johnston, L.J.; King, N.J.C. Interleukin-1β but Not Tumor Necrosis Factor Is Involved in West Nile Virus-Induced Langerhans Cell Migration from the Skin in C57BL/6 Mice. *Journal of Investigative Dermatology* **2001**, *117*, 702–709, doi:10.1046/j.0022-202x.2001.01454.x.

27. Glass, W.G.; Lim, J.K.; Cholera, R.; Pletnev, A.G.; Gao, J.L.; Murphy, P.M. Chemokine Receptor CCR5 Promotes Leukocyte Trafficking to the Brain and Survival in West Nile Virus Infection. *Journal of Experimental Medicine* **2005**, *202*, 1087–1098, doi:10.1084/jem.20042530.

28. Kumar, M.; Nerurkar, V.R. Integrated Analysis of MicroRNAs and Their Disease Related Targets in the Brain of Mice Infected with West Nile Virus. *Virology* **2014**, *0*, 143–151, doi:10.1016/j.virol.2014.01.004.

29. Lim, S.M.; van den Ham, H.J.; Oduber, M.; Martina, E.; Zaaraoui-Boutahar, F.; Roose, J.M.; van IJcken, W.F.J.; Osterhaus, A.D.M.E.; Andeweg, A.C.; Koraka, P.; et al. Transcriptomic Analyses Reveal Differential Gene Expression of Immune and Cell Death Pathways in the Brains of Mice Infected with West Nile Virus and Chikungunya Virus. *Frontiers in Microbiology* **2017**, *8*, 1556, doi:10.3389/fmicb.2017.01556.

30. Suen, W.W.; Imoda, M.; Thomas, A.W.; Nasir, N.N.B.M.; Tearnsing, N.; Wang, W.; Bielefeldt-Ohmann, H. An Acute Stress Model in New Zealand White Rabbits Exhibits Altered Immune Response to Infection with West Nile Virus. *Pathogens* **2019**, *8*, 195, doi:10.3390/pathogens8040195.

31. Maximova, O.A.; Sturdevant, D.E.; Kash, J.C.; Kanakabandi, K.; Xiao, Y.; Minai, M.; Moore, I.N.; Taubenberger, J.; Martens, C.; Cohen, J.I.; et al. Virus Infection of the Cns Disrupts the Immune-Neural-Synaptic Axis via Induction of Pleiotropic Gene Regulation of Host Responses. *Elife* **2021**, *10*, e62273, doi:10.7554/eLife.62273.

32. Clarke, P.; Leser, J.S.; Bowen, R.A.; Tylera, K.L. Virus-Induced Transcriptional Changes in the Brain Include the Differential Expression of Genes Associated with Interferon, Apoptosis, Interleukin 17 Receptor A, and Glutamate Signaling as Well as Flavivirus-Specific Upregulation of TRNA Synthetases. *mBio* **2014**, *5*, e00902-14, doi:10.1128/mBio.00902-14.

33. Brien, J.D.; Uhrlaub, J.L.; Nikolich-Zugich, J. West Nile Virus-Specific CD4 T Cells Exhibit Direct Anti-Viral Cytokine Secretion and Cytotoxicity and Are Sufficient for Antiviral Protection. *J Immunol.* **2012**, *181*, 8568–8575, doi:10.4049/jimmunol.181.12.8568.

34. Martina, B.E.E.; Koraka, P.; Doel, P. van den; Rimmelzwaan, G.F.; Haagmans, B.L.; Osterhaus, A.D.M.E. DC-SIGN Enhances Infection of Cells with Glycosylated West Nile Virus in Vitro and Virus Replication in Human Dendritic Cells Induces Production of IFN-α and TNF-α. *Virus Research* **2008**, *135*, 64–71, doi:10.1016/j.virusres.2008.02.008.

35. Zimmerman, M.G.; Bowen, J.R.; McDonald, C.E.; Pulendran, B.; Suthar, M.S. West Nile Virus Infection Blocks Inflammatory Response and T Cell Costimulatory Capacity of Human Monocyte-Derived Dendritic Cells. *J Virol* **2019**, *93*, e00664-19, doi:10.1128/ JVI.00664-19.

36. Zimmerman, M.G.; Bowen, J.R.; McDonald, C.E.; Young, E.; Baric, R.S.; Pulendran, B.; Suthar, M.S. STAT5: A Target of Antagonism by Neurotropic Flaviviruses. *Journal of Virology* **2019**, *93*, e00665-19, doi:10.1128/jvi.00665-19.

37. Suen, W.W.; Uddin, M.J.; Prow, N.A.; Bowen, R.A.; Hall, R.A.; Bielefeldt-Ohmann, H. Tissue-Specific Transcription Profile of Cytokine and Chemokine Genes Associated with Flavivirus Control and Non-Lethal Neuropathogenesis in Rabbits. *Virology* **2016**, *494*, 1–14, doi:10.1016/j.virol.2016.03.026.

38. Garcia, M.; Alout, H.; Diop, F.; Damour, A.; Bengue, M.; Weill, M.; Missé, D.; Lévêque, N.; Bodet, C. Innate Immune Response of Primary Human Keratinocytes to West Nile Virus Infection and Its Modulation by Mosquito Saliva. *Front Cell Infect Microbiol* **2018**, *8*, 387, doi:10.3389/fcimb.2018.00387.

39. Saxenaa, V.; Weltea, T.; Baob, X.; Xiea, G.; Wanga, J.; Higgsc, S.; Teshd, R.B.; Tian Wanga A Hamster-Derived West Nile Virus Strain Is Highly Attenuated and Induces a Differential Proinflammatory Cytokine Response in Two Murine Cell Lines. *Virus Res.* **2013**, *167*, 179–187, doi:10.1016/j.virusres.2012.04.013.A.

40. Fang, H.; Welte, T.; Zheng, X.; Chang, G.-J.J.; Holbrook, M.R.; Soong, L.; Tian Wang Γδ T Cells Promote the Maturation of Dendritic Cells During West Nile Virus Infection. *FEMS Immunol Med Microbiol.* **2010**, *59*, 71–80, doi:10.1111/j.1574-695X.2010.00663.x.

41. Wang, T.; Town, T.; Alexopoulou, L.; Anderson, J.F.; Fikrig, E.; Flavell, R.A. Toll-like Receptor 3 Mediates West Nile Virus Entry into the Brain Causing Lethal Encephalitis. *Nature Medicine* **2004**, *10*, 1366–1373, doi:10.1038/nm1140.

42. Uddin, M.J.; Suen, W.W.; Prow, N.A.; Hall, R.A.; Bielefeldt-Ohmann, H. West Nile Virus Challenge Alters the Transcription Profiles of Innate Immune Genes in Rabbit Peripheral Blood Mononuclear Cells. *Frontiers in Veterinary Science* **2015**, *2*, 76, doi:10.3389/fvets.2015.00076.

43. Xiea, G.; Weltea, T.; Wanga, J.; Whitemanb, M.C.; Wickerb, J.A.; Saxenaa, V.; Conga, Y.; Barretta, A.D.T.; Wang, T. A West Nile Virus NS4B-P38G Mutant Strain Induces Adaptive Immunity via TLR7-MyD88-Dependent and Independent Signaling Pathways. *Vaccine* **2013**, *31*, 4143–4151, doi:10.1016/j.vaccine.2013.06.093.A.

44. Quick, E.D.; Leser, J.S.; Clarke, P.; Tyler, K.L. Activation of Intrinsic Immune Responses and Microglial Phagocytosis in an Ex Vivo Spinal Cord Slice Culture Model of West Nile Virus Infection. *Journal of Virology* **2014**, *88*, 13005–13014, doi:10.1128/jvi.01994-14.

45. Bai, F.; Town, T.; Qian, F.; Wang, P.; Kamanaka, M.; Connolly, T.M.; Gate, D.; Montgomery, R.R.; Flavell, R.A.; Fikrig, E. IL-10 Signaling Blockade Controls Murine West Nile Virus Infection. *PLoS Pathogens* **2009**, *5*, e1000610, doi:10.1371/journal.ppat.1000610.

46. Saxena, V.; Xie, G.; Li, B.; Farris, T.; Welte, T.; Gong, B.; Boor, P.; Wu, P.; Tang, S.J.; Tesh, R.; et al. A Hamster-Derived West Nile Virus Isolate Induces Persistent Renal Infection in Mice. *PLoS Neglected Tropical Diseases* **2013**, *7*, e2275, doi:10.1371/journal.pntd.0002275.

47. Welte, T.; Reagan, K.; Fang, H.; Machain-Williams, C.; Zheng, X.; Mendell, N.; Chang, G.J.J.; Wu, P.; Blair, C.D.; Wang, T. Toll-like Receptor 7-Induced Immune Response to Cutaneous West Nile Virus Infection. *Journal of General Virology* **2009**, *90*, 2660–2668, doi:10.1099/vir.0.011783-0.

48. Luo, H.; Winkelmann, E.R.; Zhu, S.; Ru, W.; Mays, E.; Silvas, J.A.; Vollmer, L.L.; Gao, J.; Peng, B.H.; Bopp, N.E.; et al. Peli1 Facilitates Virus Replication and Promotes Neuroinflammation during West Nile Virus Infection. *Journal of Clinical Investigation* **2018**, *128*, 4980–4991, doi:10.1172/JCI99902.

49. Town, T.; Bai, F.; Wang, T.; Kaplan, A.; Qian, F.; Montgomery, R.; Anderson, J.; RA, F.; Fikrig, E. Toll-like Receptor 7 Mitigates Lethal West Nile Encephalitis via Interleukin 23-Dependent Immune Cell Infiltration and Homing. *Immunity* **2009**, *30*, 242–253, doi:10.1016/j.cortex.2009.08.003.

50. Graham, J.B.; Swarts, J.L.; Wilkins, C.; Thomas, S.; Green, R.; Sekine, A.; Voss, K.M.; Ireton, R.C.; Mooney, M.; Choonoo, G.; et al. A Mouse Model of Chronic West Nile Virus Disease. *PLoS Pathogens* **2016**, *12*, e1005996, doi:10.1371/journal.ppat.1005996.

51. Bourgeois, M.A.; Denslow, N.D.; Seino, K.S.; Barber, D.S.; Long, M.T. Gene Expression Analysis in the Thalamus and Cerebrum of Horses Experimentally Infected with West Nile Virus. *PLoS ONE* **2011**, *6*, e24371, doi:10.1371/journal.pone.0024371.

52. Acharya, D.; Wang, P.; Paul, A.; Dai, J.; Gate, D.; Lowery, J.; Stokic, D.; Leis, A.; Flavell, R.; Town, T.; et al. Interleukin-17A Promotes CD8+ T Cell Cytotoxicity To Facilitate West Nile Virus Clearance. *J Virol* **2017**, *91*, e01529-16, doi:10.1128/JVI.01529-16.

53. Wang, P.; Bai, F.; Zenewicz, L.A.; Dai, J.; Gate, D.; Cheng, G.; Yang, L.; Qian, F.; Yuan, X.; Montgomery, R.R.; et al. IL-22 Signaling Contributes to West Nile Encephalitis Pathogenesis. *PLoS ONE* **2012**, *7*, e44153, doi:10.1371/journal.pone.0044153.

54. Bryan, M.A.; Giordano, D.; Draves, K.E.; Green, R.; Gale, M.; Clark, E.A. Splenic Macrophages Are Required for Protective Innate Immunity against West Nile Virus. *PLoS ONE* **2018**, *13*, e0191690, doi:10.1371/journal.pone.0191690.

Supplementary table S2: Chemokines involved in West Nile virus pathogenesis.

Cytokine	Mode	Host	Expression	Source	Role in WNV pathogenesis	Reference
	**CC- Chemokines**					
**CCL1**	*In vivo*	Mouse	Downregulation	Brain	Unknown	[1]
**CCL2**	*In vitro*	Human	No change	LAN-2/ U-937/ MΦ/ BMDCs/ BMECs/ astrocytes	Unknown	[2–4]
			Upregulation	Microglia/ U373/ ARPE-19/RPE	Unknown	[2,5,6]
			Strain-dependent	SK-N-SH cells.	Unknown	[7]
		Mouse	Upregulation	Microglia/ neurons	Unknown	[8,9]
	In vivo	Mouse	-	-	Mediates monocytes migration and differentiation into DC in the skin and auricular lymph node	[10]
			Upregulation	Serum/ spleen/ lung/ liver/ brain	Not required for survival/ neutralization enhances survival<br>Participates in monocytosis and monocytes trafficking to the brain	[1,9,11–20]
				Neurons/ astrocytes/ microglia/ inflammatory cells/ endothelial cells	Unknown	[21]
			Strain-dependent	Brain	Unknown	[22]
		NHP (*Macaca mulatta)*	Upregulation	Brain/spinal cord	Unknown	[23]
	In vivo	Mouse	Upregulation	Spinal cord slice	Unknown	[24]
**CCL3**	*In vitro*	Human	No change	BMDCs	Unknown	[3]
	In vivo	Mouse	Upregulation	Serum/ brain/spleen	Unknown	[1,9,11,12,14–16,25]
			Strain-dependent	Brain	Unknown	[22]
		NHP	Upregulation	Brain/spinal cord	Unknown	[23]

		(*Macaca mulatta)*				
	In vivo	Mouse	Upregulation	Spinal cord slice	Unknown	[24]
**CCL4**	*In vitro*	Human	No change	BMDCs	Unknown	[3]
			Upregulation	NK cells	Unknown	[26]
		Mouse	Upregulation	Microglia	Unknown	[8]
	In vivo	Mouse	Upregulation	Brain/ spleen/ lung	Unknown	[9,11–14,16,25]
			Strain-dependent	Brain	Unknown	[22]
**CCL5**	*In vitro*	Human	No change	BMDCs	Unknown	[3]
			Upregulation	Astrocytes/ Microglia/ ARPE-19/RPE/ SK-N-SH/ MΦ/ BMECs	Unknown	[4–7,27]
			Strain-dependent	THP-1/ THP-1-derived MΦ	Unknown	[28]
		Mouse	Upregulation	MΦ/ neurons	Unknown	[9,29]
	In vivo	Mouse	Upregulation	Serum/brain/spleen/ lung	Unknown	[1,9,11–16,25]
				Astroglia	Unknown	[21]
			Strain-dependent	Brain	Unknown	[22]
		NHP (*Macaca mulatta)*	Upregulation	Brain/spinal cord	Unknown	[23]
		Rabbit (*Oryctolagus cuniculus*)	Upregulation	Draining lymph nodes	Unknown	[30]
	In vivo	Mouse	Upregulation	Spinal cord slice	Unknown	[24]
**CCL6**[1]	*In vivo*	Mouse	Upregulation	Brain	Unknown	[13]
**CCL7**	*In vivo*	Mouse	Upregulation	Blood/ spleen/ lung/ liver/ brain	Required for efficient monocytosis, recruitment of neutrophils and CD8+ T cells into the CNS, survival after lethal challenge and viral clearance from the brain, not blood or spleen.	[1,9,11,15,17,18,20]
			Strain-dependent	Brain	Unknown	[22]

**CCL8**	*In vitro*	Human	Upregulation	MΦ	Unknown	[27]
	In vivo	Mouse	Upregulation	Brain	Unknown	[1]
			Strain-dependent	Brain	Unknown	[22]
		NHP (*Macaca mulatta)*	Upregulation	Brain/spinal cord	Unknown	[23]
**CCL9**	*In vivo*	Mouse	Upregulation	Brain	Unknown	[1]
**CCL11**	*In vivo*	Mouse	Upregulation	Lung/ Brain	Unknown	[11,12]
**CCL12**[1]	*In vivo*	Mouse	Upregulation	Brain	Unknown	[1,13,18]
**CCL13**	*In vivo*	Mouse	Upregulation	Brain	Unknown	[16]
**CCL15**	*In vivo*	Rabbit (*Oryctolagus cuniculus*)	Downregulation	Draining lymph nodes	Unknown	[30]
**CCL17**	*In vivo*	Mouse	Upregulation	Lung	Unknown	[11]
		Rabbit (*Oryctolagus cuniculus*)	Downregulation	Draining lymph nodes	Unknown	[30]
**CCL18**	*In vivo*	Mouse	Upregulation	Brain	Unknown	[18]
**CCL19**	*In vivo*	Mouse	No change	Brain	Unknown	[9]
			Upregulation	Spleen/ lung/ brain	Unknown	[1,11]
			Strain-dependent	Brain	Unknown	[22]
**CCL20**	*In vitro*	Human	Upregulation	ARPE-19/RPE/ keratinocytes	Unknown	[6,31]
	In vivo	Mouse	Downregulation	Brain	Unknown	[1]
**CCL21**	*In vivo*	Mouse	No change	Brain	Unknown	[9]
			Upregulation	Lung	Unknown	[11]
		Rabbit (*Oryctolagus cuniculus*)	Downregulation	Draining lymph nodes	Unknown	[30]
**CCL22**	*In vivo*	Mouse	Upregulation	Lung/kidney	Unknown	[11]
**CCL24**	*In vivo*	Mouse	Upregulation	Spleen/ lung/ liver	Unknown	[11]
			Downregulation	Brain	Unknown	[1]
	**CC- Chemokines receptors**					

**Ccr1**	*In vivo*	Mouse	Upregulation	Lung/brain	Unknown	[10, 14]14]
**Ccr2**	*In vivo*	Mouse	Upregulation	Lung/brain	Protects from lethal challenge and promotes viral clearance from the CNS.<br>Required for monocytosis and accumulation of monocytes in the brain by regulating blood monocyte levels, not trafficking from blood to brain.	[1,11,18]
**Ccr3**	*In vitro*	Human	Upregulation	MΦ	Restricts viral replication	[27]
	In vivo	Mouse	Upregulation	Brain	Unknown	[1]
**Ccr4**	*In vivo*	Mouse	Downregulation	Brain	Unknown	[1]
		Rabbit (*Oryctolagus cuniculus*)	Downregulation	Draining lymph nodes	Unknown	[30]
**Ccr5**	*In vivo*	Mouse	-	-	Enhances survival following WNV challenge<br>Controls viral loads in the brain and the BBB permeability	[32]
			-	CD4+ and CD8+ T cells, NK, MΦ	Crucial for survival and viral clearance in the brain not the spleen<br>Promotes leukocyte trafficking to the CNS	[14]
			Upregulation	Brain	Unknown	[14,18]
		Rabbit (*Oryctolagus cuniculus*)	Upregulation	Draining lymph nodes	Unknown	[30]
**Ccr6**	*In vivo*	Mouse	Downregulation	Brain	Unknown	[1]
**Ccr7**	*In vivo*	Mouse	-	Brain	Required for survival<br>DC and T cell trafficking to the lymph nodes<br>Does not impact leukocyte accumulation or T cell priming in the spleen<br>Limits the infiltration of WNV-infected myeloid cells into the CNS.	[14,32][14,
**Ccr8**	*In vivo*	Mouse	Downregulation	Brain	Unknown	[1]
		Rabbit (*Oryctolagus	Downregulation	Draining lymph nodes	Unknown	[30]

		cuniculus)				
**Ccr9**	*In vivo*	Mouse	Upregulation	Brain	Unknown	[1]
	**CX-Chemokines**					
**CXCL1**	*In vitro*	Human	Upregulation	MΦ/ keratinocytes	Unknown	[31,34]
		Mouse	Upregulation	Neutrophils/ MΦ	Unknown	[34]
	In vivo	Mouse	Upregulation	Serum/ brain	Unknown	[1,12,14,20]
			Strain-dependent	Brain	Unknown	[22]
		NHP (*Macaca mulatta)*	Upregulation	Brain/spinal cord	Unknown	[23]
	Ex vivo	Mouse	Upregulation	Spinal cord slice	Unknown	[24]
**CXCL2**	*In vitro*	Human	Upregulation	MΦ/ keratinocytes	Unknown	[31,34]
		Mouse	Upregulation	Neutrophils/ MΦ	Unknown	[34]
	In vivo	Mouse	Upregulation	Lung/ liver/ brain	Unknown	[11–14,16,20]
**CXCL3**	*In vivo*	Mouse	Upregulation	Spleen/ kidney	Unknown	[11]
**CXCL4**	*In vivo*	Mouse	Upregulation	Brain	Unknown	[14]
**CXCL5**	*In vivo*	Mouse	Upregulation	Spleen/ brain	Unknown	[11,12]
**CXCL8/IL-8**[2]	*In vitro*	Human	No change	Activated MΦ	Unknown	[35]
			Upregulation	MΦ/ SK-N-SH/ ARPE-19/RPE/ keratinocytes/ BMDCs/ plasmacytoid DCs	Not responsible for WNV-induced cell death in SK-N-SH	[6,27,31,35–37]
			Strain-dependent	SK-N-SH	Unknown	[7]
	In vivo	NHP (*Macaca mulatta)*	Upregulation	Brain/spinal cord	Unknown	[23]
**CXCL9**	*In vitro*	Human	No change	BMDCs	Unknown	[3]
		Mouse	Upregulation	Neurons	Unknown	[9]
	In vivo	Mouse	-	-	Not required for survival after lethal challenge	[38]
			Upregulation	Spleen/ lung/ liver/ brain	Unknown	[9,11–16,20]
		Horse	Upregulation	Thalamus/ cerebrum	Unknown	[39]
		Rabbit	Upregulation	Draining lymph nodes	Unknown	[30]

		(*Oryctolagus cuniculus*)				
**CXCL10/IP10**	*In vitro*	Human	Upregulation	Astrocytes/ Microglia/ THP-1-derived MΦ/ MΦ/ LAN-2/ PBMCs/U373/ U-937/ ARPE-19/RPE/ SK-N-SH/ keratinocytes/ BMDCs/ Plasmacytoid DCs	Unknown	[2,5–7,27,28,31,34,37,40]
			Strain-dependent	THP-1	Unknown	[28]
		Mouse	Upregulation	Neutrophils/ MΦ/ microglia	Unknown	[8,29,34]
		Rabbit (*Oryctolagus cuniculus*)	Upregulation	Draining Lymph nodes/ PBMCs	Unknown	[30,41]
	In vivo	Mouse	Upregulation	Serum/ spleen/ lung/ liver/ brain	Required for T-lymphocyte recruitment into the CNS, control of viral infection, and survival after WNV lethal challenge	[1,9,11–16,20,25,38,42]
				Neurons/ neutrophils/ astroglia	Unknown	[21]
			Strain-dependent	Brain	Unknown	[22]
		Rabbit (*Oryctolagus cuniculus* and *Sylvilagus sp)*	Upregulation	Spleen/LNs/ Brain	Unknown	[43]
		**NHP (*Macaca mulatta)***	Upregulation	Brain/spinal cord	Unknown	[23]
		Horse	Upregulation	Lymphoid tissue/ brain	Unknown	[44]
	In vivo	Mouse	Upregulation	Spinal cord slice	Unknown	[24]
**CXCL11**	*In vitro*	Mouse	Upregulation	Neurons	Unknown	[9]
	In vivo	Mouse	Upregulation	Brain	Unknown	[1,15]
		NHP	Upregulation	Brain/spinal cord	Unknown	[23]

		(*Macaca mulatta)*				
		Rabbit (*Oryctolagus cuniculus*)	Upregulation	Draining lymph nodes	Unknown	[30]
**CXCL12**	*In vitro*	Mouse	-	BMECs	IL-1β mediates the ability of Cxcr4 expressing lymphocytes to adhere to BMECs via CXCL12 expression on their basolateral surface	[45]
	In vivo	Mouse	No change	Brain	Unknown	[20,46]
			Downregulation (β isoform)	Brain	Unknown	[45,46]
			Upregulation	Brain	Unknown	[22]
	In vivo	Mouse	Upregulation	Primary BMECs and astrocytes	CXCL12 expression at the CNS microvasculature is IL-1- mediated and regulates the efficient parenchymal entry of T lymphocytes.	[45]
**CXCL13**	*In vivo*	Mouse	No change	Brain/spleen	Unknown	[25]
			Upregulation	Brain	Unknown	[1,15]
			Strain-dependent	Brain	Unknown	[22]
		NHP (*Macaca mulatta)*	Upregulation	Brain/spinal cord	Unknown	[23]
**CXCL14**	*In vivo*	Mouse	Upregulation	Brain	Unknown	[25]
			Strain dependent	Spleen	Unknown	[25]
**CXCL15**	*In vivo*	Mouse	Downregulation	Brain	Unknown	[1]
**CXCL16**	*In vivo*	Mouse	Strain-dependent	Brain	Unknown	[22]
	**CX-Chemokines receptors**					
**Cxcr2**	*In vivo*	Mouse	-	-	Enhances viremia and death	[34]
**Cxcr3**	*In vitro*	Human	Upregulation	MΦ	Restricts viral replication	[27]
		Mouse	No change	Microglia	Unknown	[47]

			Downregulation	Neurons	Does not influence viral replication. Leads to cell death at early time-points. Expression on neurons via TNFR1 signaling.	[47]
	In vivo	Mouse	-	-	Required for survival after lethal WNV-challenge and for CD8+ T cells control of WNV infection within the cerebellum.	[38]
			Downregulation during early phase	Neurons/ CD8+ T cells	Unknown	[47]
			Upregulation	Brain	Unknown	[14,18]
**Cxcr4**	*In vivo*	Mouse	-	Macrophage/ microglia/ CD8+ T cells	Enhances mortality and viral loads in the brain via downregulating T cells trafficking Increases glial cells activation	[46]
			Upregulation	Lung/ brain	Unknown	[11,14,16]
**CX3C Chemokine and its receptor**						
**CX3CL1**	*In vivo*	Mouse	Upregulation	Brain (microglia/monocytes)	Does not impact survival following WNV challenge. Monocytes (microglial precursor) recruitment to the brain.	[14]
		NHP (*Macaca mulatta)*	Upregulation	Spinal cord	Unknown	[23]
**Cx3cr1**	*In vivo*	Mouse	Upregulation	-	Does not impact survival following WNV challenge.	[14,19,33]
		NHP (*Macaca mulatta)*	Downregulation	Cerebrum/ spinal cord	Unknown	[23]
**XC chemokine receptor**						
**Xcr1**	*In vivo*	Mouse	Downregulation	Brain	Unknown	[1]

[1] Present only in mice

[2] Present only in humans

Cell Lines: H36.12j; *Mus musculus* (macrophage cell line) ATCC CRL-2449™; Renca *Mus musculus* (Kidney epithelial cells line) ATCC CRL-2947 ™; retinal pigment epithelium cell line (ARPE-19): L929 ATCC CCL-1™; LAN-2: *Mus musculus* neuroblastoma cell line (RRID:CVCL_1829); U-937: *Homo sapiens* Lymphoma cell line CRL-1593.2 ™; U373: *Homo sapiens* glioblastoma astrocytoma cell-line; SK-N-SH *Homo sapiens* neuroblastoma cell line ATCC HTB-11™; THP-1: Monocytic leukemia cells ATCC TIB-202™.

Abbreviations: ASC: Apoptosis-associated speck-like protein containing C-terminal caspase recruitment domain [CARD]; BBB: Blood-brain barrier; BMDCs: Bone marrow-derived dendritic cells; BMECs: Brain microvasculature endothelial cells; BMECs: Brain microvascular endothelial cells; CNS: Central nervous system; DCs: Dendritic cells; NHP: Non-Human primate; RPE: retinal pigment epithelium (RPE); IFN: Interferon, IRF: IFN regulatory factor 3; MΦ: Macrophages; PBMCs: Peripheral blood mononuclear cells.

References:

1. Kumar, M.; Nerurkar, V.R. Integrated Analysis of MicroRNAs and Their Disease Related Targets in the Brain of Mice Infected with West Nile Virus. *Virology* **2014**, *0*, 143–151, doi:10.1016/j.virol.2014.01.004.

2. Marle, G. Van; Antony, J.; Ostermann, H.; Dunham, C.; Hunt, T.; Halliday, W.; Maingat, F.; Urbanowski, M.D.; Hobman, T.; Peeling, J.; et al. West Nile Virus-Induced Neuroinflammation : Glial Infection and Capsid Protein-Mediated Neurovirulence. *J Virol* **2007**, *81*, 10933–10949, doi:10.1128/JVI.02422-06.

3. Zimmerman, M.G.; Bowen, J.R.; McDonald, C.E.; Pulendran, B.; Suthar, M.S. West Nile Virus Infection Blocks Inflammatory Response and T Cell Costimulatory Capacity of Human Monocyte-Derived Dendritic Cells. *J Virol* **2019**, *93*, e00664-19, doi:10.1128/ JVI.00664-19.

4. Hussmann, K.L.; Fredericksen, B.L. Differential Induction of CCL5 by Pathogenic and Non-Pathogenic Strains of West Nile Virus in Brain Endothelial Cells and Astrocytes. *Journal of General Virology* **2014**, *95*, 862–867, doi:10.1099/vir.0.060558-0.

5. Cheeran, M.C.J.; Hu, S.; Sheng, W.S.; Rashid, A.; Peterson, P.K.; Lokensgard, J.R. Differential Responses of Human Brain Cells to West Nile Virus Infection. *Journal of NeuroVirology* **2005**, *11*, 512–524, doi:10.1080/13550280500384982.

6. Munoz-erazo, L.; Natoli, R.; Provis, J.M.; Madigan, M.C.; Jonathan, N.; King, C. Microarray Analysis of Gene Expression in West Nile Virus – Infected Human Retinal Pigment Epithelium. *Molecular Vision* **2012**, *18*, 730–743.

7. Huang, B.; West, N.; Vider, J.; Zhang, P.; Griffiths, R.E.; Wolvetang, E.; Burtonclay, P.; Warrilow, D. Inflammatory Responses to a Pathogenic West Nile Virus Strain. *BMC Infectious Diseases* **2019**, *19*, 912, doi:10.1186/s12879-019-4471-8.

8. Luo, H.; Winkelmann, E.R.; Zhu, S.; Ru, W.; Mays, E.; Silvas, J.A.; Vollmer, L.L.; Gao, J.; Peng, B.H.; Bopp, N.E.; et al. Peli1 Facilitates Virus Replication and Promotes Neuroinflammation during West Nile Virus Infection. *Journal of Clinical Investigation* **2018**, *128*, 4980–4991, doi:10.1172/JCI99902.

9. Klein, R.S.; Lin, E.; Zhang, B.; Luster, A.D.; Tollett, J.; Samuel, M.A.; Engle, M.; Diamond, M.S. Neuronal CXCL10 Directs CD8 + T-Cell Recruitment and Control of West Nile Virus Encephalitis . *Journal of Virology* **2005**, *79*, 11457–11466, doi:10.1128/jvi.79.17.11457-11466.2005.

10. Davison, A.M.; Nicholas J C King Accelerated Dendritic Cell Differentiation from Migrating Ly6C Lo Bone Marrow Monocytes in Early Dermal West Nile Virus Infection. *Journal of immunology* **2011**, *186*, 2382–2396, doi:10.4049/jimmunol.1002682.

11. Peña, J.; Plante, J.A.; Carillo, A.C.; Roberts, K.K.; Smith, J.K.; Juelich, T.L.; Beasley, D.W.C.; Freiberg, A.N.; Labute, M.X.; Naraghi-Arani, P. Multiplexed Digital MRNA Profiling of the Inflammatory Response in the West Nile Swiss Webster Mouse Model. *PLoS Neglected Tropical Diseases* **2014**, *8*, e3216, doi:10.1371/journal.pntd.0003216.

12. Kumar, M.; Roe, K.; O'Connell, M.; Nerurkar, V.R. Induction of Virus-Specific Effector Immune Cell Response Limits Virus Replication and Severe Disease in Mice Infected with Non-Lethal West Nile Virus Eg101 Strain. *Journal of Neuroinflammation* **2015**, *12*, 178, doi:10.1186/s12974-015-0400-y.

13. Garcia-tapia, D.; Hassett, D.E.; Mitchell Jr, W.J.; Johnson, G.C.; Kleiboeker, S.B. West Nile Virus Encephalitis : Sequential Histopathological and Immunological Events in a Murine Model of Infection. *Journal of NeuroVirology* **2007**, *13*, 130–138, doi:10.1080/13550280601187185.

14. Glass, W.G.; Lim, J.K.; Cholera, R.; Pletnev, A.G.; Gao, J.L.; Murphy, P.M. Chemokine Receptor CCR5 Promotes Leukocyte Trafficking to the Brain and Survival in West Nile Virus Infection. *Journal of Experimental Medicine* **2005**, *202*, 1087–1098, doi:10.1084/jem.20042530.

15. Clarke, P.; Leser, J.S.; Bowen, R.A.; Tylera, K.L. Virus-Induced Transcriptional Changes in the Brain Include the Differential Expression of Genes Associated with Interferon, Apoptosis, Interleukin 17 Receptor A, and Glutamate Signaling as Well as Flavivirus-Specific Upregulation of TRNA Synthetases. *mBio* **2014**, *5*, e00902-14, doi:10.1128/mBio.00902-14.

16. Lim, S.M.; van den Ham, H.J.; Oduber, M.; Martina, E.; Zaaraoui-Boutahar, F.; Roose, J.M.; van IJcken, W.F.J.; Osterhaus, A.D.M.E.; Andeweg, A.C.; Koraka, P.; et al. Transcriptomic Analyses Reveal Differential Gene Expression of Immune and Cell Death Pathways in the Brains of Mice Infected with West Nile Virus and Chikungunya Virus. *Frontiers in Microbiology* **2017**, *8*, 1556, doi:10.3389/fmicb.2017.01556.

17. Bardina, S. v; Michlmay, D.; Hoffman, K.W.; Obara, C.J.; Sum, J.; Charo, I.F.; Lu, W.; Pletnev, A.G.; Lim, J.K. Differential Roles of Chemokines CCL2 and CCL7 in Monocytosis and Leukocyte Migration during West Nile Virus Infection. *Physiol Behav* **2015**, *195*, 4306–4318, doi:10.4049/jimmunol.1500352.

18. Lim, J.K.; Obara, C.J.; Rivollier, A.; Pletnev, A.G.; Kelsall, B.L.; Murphy, P.M. Chemokine Receptor CCR2 Is Critical for Monocyte Accumulation and Survival in West Nile Virus Encephalitis. *J Immunol* **2011**, *186*, 471–478, doi:10.4049/jimmunol.1003003.

19. Getts, D.R.; Terry, R.L.; Getts, M.T.; Marcus, M.; Rana, S.; Shrestha, B.; Radford, J.; Rooijen, N. van; Campbell, I.L.; King, N.J.C. Ly6c+ " Inflammatory Monocytes " Are Microglial Precursors Recruited in a Pathogenic Manner in West Nile Virus Encephalitis. *J. Exp. Med* **2008**, *205*, 2319–2337, doi:10.1084/jem.20080421.

20. Michlmayr, D.; McKimmie, C.S.; Pingen, M.; Haxton, B.; Mansfield, K.; Johnson, N.; Fooks, A.R.; Graham, G.J. Defining the Chemokine Basis for Leukocyte Recruitment during Viral Encephalitis. *Journal of Virology* **2014**, *88*, 9553–9567, doi:10.1128/jvi.03421-13.

21. Vidaña, B.; Johnson, N.; Fooks, A.R.; Sánchez-Cordón, P.J.; Hicks, D.J.; Nuñez, A. West Nile Virus Spread and Differential Chemokine Response in the Central Nervous System of Mice: Role in Pathogenic Mechanisms of Encephalitis. *Transbound Emerg Dis* **2020**, *67*, 799–810, doi:10.1111/tbed.13401.

22. Kumar, M.; Belcaid, M.; Nerurkar, V.R. Identification of Host Genes Leading to West Nile Virus Encephalitis in Mice Brain Using RNA-Seq Analysis. *Scientific Reports* **2016**, *6*, 26350, doi:10.1038/srep26350.

23. Maximova, O.A.; Sturdevant, D.E.; Kash, J.C.; Kanakabandi, K.; Xiao, Y.; Minai, M.; Moore, I.N.; Taubenberger, J.; Martens, C.; Cohen, J.I.; et al. Virus Infection of the Cns Disrupts the Immune-Neural-Synaptic Axis via Induction of Pleiotropic Gene Regulation of Host Responses. *Elife* **2021**, *10*, e62273, doi:10.7554/eLife.62273.

24. Quick, E.D.; Leser, J.S.; Clarke, P.; Tyler, K.L. Activation of Intrinsic Immune Responses and Microglial Phagocytosis in an Ex Vivo Spinal Cord Slice Culture Model of West Nile Virus Infection. *Journal of Virology* **2014**, *88*, 13005–13014, doi:10.1128/jvi.01994-14.

25. Shirato, K.; Kimura, T.; Mizutani, T.; Kariwa, H.; Takashima, I. Different Chemokine Expression in Lethal and Non-Lethal Murine West Nile Virus Infection. *Journal of Medical Virology* **2004**, *74*, 507–513, doi:10.1002/jmv.20205.

26. Yao, Y.; Strauss-albee, D.M.; Zhou, J.Q.; Malawista, A.; Garcia, M.N.; Murray, K.O.; Blish, C.A.; Montgomery, R.R. The Natural Killer Cell Response to West Nile Virus in Young and Old Individuals with or without a Prior History of Infection. *PLoS ONE* **2017**, *12*, e0172625, doi:10.1371/journal.pone.0172625.

27. Qian, F.; Chung, L.; Zheng, W.; Bruno, V.; Alexander, R.P.; Wang, Z.; Wang, X.; Kurscheid, S.; Zhao, H.; Fikrig, E.; et al. Identification of Genes Critical for Resistance to Infection by West Nile Virus Using RNA-Seq Analysis. *Viruses* **2013**, *5*, 1664–1681, doi:10.3390/v5071664.

28. Xie, G.; Luo, H.; Tian, B.; Mann, B.; Bao, X.; Mcbride, J.; Tesh, R.; Barrett, A.D.; Wang, T. A West Nile Virus NS4B-P38G Mutant Strain Induces Cell Intrinsic Innate Cytokine Responses in Human Monocytic and Macrophage Cells. *Vaccine* **2015**, *33*, 869–878, doi:10.1016/j.vaccine.2014.12.056.A.

29. Shirato, K.; Miyoshi, H.; Kariwa, H.; Takashima, I. The Kinetics of Proinflammatory Cytokines in Murine Peritoneal Macrophages Infected with Envelope Protein-Glycosylated or Non-Glycosylated West Nile Virus. *Virus Research* **2006**, *121*, 11–16, doi:10.1016/j.virusres.2006.03.010.

30. Suen, W.W.; Imoda, M.; Thomas, A.W.; Nasir, N.N.B.M.; Tearnsing, N.; Wang, W.; Bielefeldt-Ohmann, H. An Acute Stress Model in New Zealand White Rabbits Exhibits Altered Immune Response to Infection with West Nile Virus. *Pathogens* **2019**, *8*, 195, doi:10.3390/pathogens8040195.

31. Garcia, M.; Alout, H.; Diop, F.; Damour, A.; Bengue, M.; Weill, M.; Missé, D.; Lévêque, N.; Bodet, C. Innate Immune Response of Primary Human Keratinocytes to West Nile Virus Infection and Its Modulation by Mosquito Saliva. *Front Cell Infect Microbiol* **2018**, *8*, 387, doi:10.3389/fcimb.2018.00387.

32. Durrant, D.M.; Daniels, B.P.; Pasieka, T.; Dorsey, D.; Klein, R.S. CCR5 Limits Cortical Viral Loads during West Nile Virus Infection of the Central Nervous System. *Journal of Neuroinflammation* **2015**, *12*, 233, doi:10.1186/s12974-015-0447-9.

33. Bardina, S. v; Brown, J.A.; Michlmayr, D.; Hoffman, K.W. Chemokine Receptor Ccr7 Restricts Fatal West Nile Virus Encephalitis. *J Virol* **2017**, *91*, e02409-16, doi:10.1128/JVI.02409-16.

34. Bai, F.; Kong, K.; Dai, J.; Qian, F.; Zhang, L.; Brown, C.R.; Fikrig, E.; Montgomery, R.R. A Paradoxical Role for Neutrophils in the Pathogenesis of West Nile Virus. *Journal of Infectious Diseases* **2010**, *8031*, 1804–1812, doi:10.1086/657416.

35. Kong, K.; Wang, X.; Anderson, J.; Fikrig, E.; RR Montgomery West Nile Virus Attenuates Activation of Primary Human Macrophages. *Viral Immunol* **2008**, *21*, 78–82, doi:10.1089/vim.2007.0072.

36. Kumar, M.; Verma, S.; Nerurkar, V.R. Pro-Inflammatory Cytokines Derived from West Nile Virus (WNV)-Infected SK-N-SH Cells Mediate Neuroinflammatory Markers and Neuronal Death. *Journal of Neuroinflammation* **2010**, *7*, 73, doi:10.1186/1742-2094-7-73.

37. Silva, M.C.; Guerrero-plata, A.; Gilfoy, F.D.; Garofalo, R.P.; Mason, P.W. Differential Activation of Human Monocyte-Derived and Plasmacytoid Dendritic Cells by West Nile Virus Generated in Different Host Cells □. *Journal of Virology* **2007**, *81*, 13640–13648, doi:10.1128/JVI.00857-07.

38. Zhang, B.; Chan, Y.K.; Lu, B.; Diamond, M.S.; Klein, R.S. CXCR3 Mediates Region-Specific Antiviral T Cell Trafficking within the Central Nervous System during West Nile Virus Encephalitis. *The Journal of Immunology* **2008**, *180*, 2641–2649, doi:10.4049/jimmunol.180.4.2641.

39. Bourgeois, M.A.; Denslow, N.D.; Seino, K.S.; Barber, D.S.; Long, M.T. Gene Expression Analysis in the Thalamus and Cerebrum of Horses Experimentally Infected with West Nile Virus. *PLoS ONE* **2011**, *6*, e24371, doi:10.1371/journal.pone.0024371.

40. Qian, F.; Goel, G.; Meng, H.; Wang, X.; You, F.; Devine, L.; Raddassi, K.; Garcia, M.N.; Murray, K.O.; Bolen, C.R.; et al. Systems Immunology Reveals Markers of Susceptibility to West Nile Virus Infection. *Clin Vaccine Immunol* **2015**, *22*, 6–16, doi:10.1128/CVI.00508-14.

41. Uddin, M.J.; Suen, W.W.; Prow, N.A.; Hall, R.A.; Bielefeldt-Ohmann, H. West Nile Virus Challenge Alters the Transcription Profiles of Innate Immune Genes in Rabbit Peripheral Blood Mononuclear Cells. *Frontiers in Veterinary Science* **2015**, *2*, 76, doi:10.3389/fvets.2015.00076.

42. Suthar, M.S.; Brassil, M.M.; Blahnik, G.; McMillan, A.; Ramos, H.J.; Proll, S.C.; Belisle, S.E.; Katze, M.G.; Gale, M. A Systems Biology Approach Reveals That Tissue Tropism to West Nile Virus Is Regulated by Antiviral Genes and Innate Immune Cellular Processes. *PLoS Pathogens* **2013**, *9*, e1003168, doi:10.1371/journal.ppat.1003168.

43. Suen, W.W.; Uddin, M.J.; Prow, N.A.; Bowen, R.A.; Hall, R.A.; Bielefeldt-Ohmann, H. Tissue-Specific Transcription Profile of Cytokine and Chemokine Genes Associated with Flavivirus Control and Non-Lethal Neuropathogenesis in Rabbits. *Virology* **2016**, *494*, 1–14, doi:10.1016/j.virol.2016.03.026.

44. Bielefeldt-ohmann, H.; Bosco-lauth, A.; Hartwig, A.; Uddin, M.J.; Barcelon, J.; Suen, W.W.; Wang, W.; Hall, R.A.; Bowen, R.A. Microbial Pathogenesis Characterization of Non-Lethal West Nile Virus (WNV) Infection in Horses : Subclinical Pathology and Innate Immune Response. *Microbial Pathogenesis* **2017**, *103*, 71–79, doi:10.1016/j.micpath.2016.12.018.

45. Durrant, D.M.; Daniels, B.P.; Klein, R.S. IL-1R1 Signaling Regulates CXCL12-Mediated T Cell Localization and Fate within the CNS during West Nile Virus Encephalitis. *J Immunol* **2014**, *193*, 4095–4106, doi:10.4049/jimmunol.1401192.

46. McCandless, E.E.; Zhang, B.; Diamond, M.S.; Klein, R.S. CXCR4 Antagonism Increases T Cell Trafficking in the Central Nervous System and Improves Survival from West Nile Virus Encephalitis. *Proc Natl Acad Sci U S A* **2008**, *105*, 11270–11275, doi:10.1073/pnas.0800898105.

47. Zhang, B.; Patel, J.; Croyle, M.; Diamond, M.S.; Klein, R.S. TNF-α-Dependent Regulation of CXCR3 Expression Modulates Neuronal Survival during West Nile Virus Encephalitis. *Journal of Neuroimmunology* **2010**, *224*, 28–38, doi:10.1016/j.jneuroim.2010.05.003.

Supplementary table S3: Tumor necrosis factor superfamily ligands involved in West Nile virus pathogenesis.

Cytokine	Model	Host	Expression	Source	Role in WNV pathogenesis	Reference
**TNF- α**	*In vitro*	Human	No change	Astrocytes/	Unknown	[1]
			Upregulation	Microglia/ Neural stem cells/ BMDCs/ SK-N-SH/ MΦ/ ARPE-19/RPE/ keratinocytes	WNV infection induces antiviral indoleamine via TNFα.<br>Neurotoxic	[1–9]
			Strain-dependent	THP-1/ THP-1-derived MΦ	Unknown	[10]
		Pig (*Sus domesticus*)	Upregulation	BMDCs	Unknown	[3]
		Mouse	Upregulation	Splenic and brain CD8+ T cell /neurons/ MΦ/ DCs/ H36.12j/ Renca/ γδ T cells/ microglia	Downregulates CXCR3 expression on neurons via TNFR1 signaling.<br>Enhances neurons survival at early time-points.<br>Reduces viral replication in MΦ and fibroblasts, not neurons and DCs.<br>Not required to upregulate MHC-I in fibroblasts/ Does not activate NF-κB during WNV infection of fibroblasts	[11–17]
				Peritoneal exudate cells	Expression may be induced by WNV envelope protein	[18]
			Strain-dependent	MΦ	Unknown	[19]
			-	Microglia/ N2a and primary cultures of murine cortical neurons	Production in microglia mediated by TLR3.<br>Direct neuronal injury.	[20]
	In vivo	Mouse	Upregulation	Blood/liver/ spleen/ kidney/ brain	Variable effects on viral replication and survival after WNV challenge.<br>Controls leukocyte infiltration in the CNS/ down-regulates neuronal CXCR3 and subsequent neuronal apoptosis/ enhances BBB permeability.<br>Expression positively regulated by SARM and TLR3 in the brain.<br>Does not affect the early antibody responses and CD8+ T cells priming in the spleen after WNV infection.	[13,18,20–29]

			-	-	TNF-/- mice developed severe limbic seizures like wild type mice.<br>Does not affect Langerhans cell migration and accumulation in the draining lymph nodes and viral entry into the CNS	[30–32]
			Strain-dependent	Brain	Unknown	[33]
		Rabbit (*Oryctolagus cuniculus* and *Sylvilagus sp.)*	Upregulation	PBMCs/ brain/ lymph nodes	Unknown	[34,35]
		Horse	Upregulation	Lymphoid tissue	Unknown	[36]
		NHP (*Macaca mulatta)*	Upregulation	Brain/spinal cord	Unknown	[37]
	In vivo	Mouse	Upregulation	Spinal cord slice	Unknown	[38]
**TRAIL**	***In vitro***	Mouse	-	Cortical neurons	CD8+ T cells require TRAIL to optimally control WNV infection in neurons.	[39]
			Negatively correlated to replication rate	L929	Unknown	[40]
	In vivo	Mouse	-	-	Protected mice from lethal infection and enhanced viral clearance from the CNS.<br>Reduced viral replication in the CNS and not in the serum or spleen when footpad- infected, not intracranially-infected.<br>Did not affect trafficking of leukocytes to the brain neither adaptive B nor T cell responses induction after WNV infection.	[39]
			Upregulation	Brain	Unknown	[28]
			Strain-dependent	Brain	Unknown	[33]
			Negatively correlated to replication rate	L929	Unknown	[40]
		Rabbit (*Oryctolagus cuniculus*)	Upregulation	Draining lymph nodes	Unknown	[30]
	In vivo	Mouse	Upregulation	Spinal cord slice	Unknown	[38]

FasL	*In vitro*	Mouse	Upregulation	Cortical neurons	Used by CD8+ T cells to control neuronal infection.	[41]
	In vivo	Mouse	Upregulation	Lung/brain	Protects mice from lethal WNV infection Required for the clearance of WNV infection from the CNS	[23,33,41]
			-	-	Does not protect mice from lethal infection	[42]
		NHP (*Macaca mulatta)*	Upregulation	Brain/spinal cord	Unknown	[37]
**CD40L**	*In vivo*	Mouse	-	-	Protects from lethal WNV infection Required for efficient production of neutralizing antibodies, trafficking of CD8+ T cells into the brain, and control of WNV replication in the CNS not the serum and spleen.	[43]
			Upregulation	Brain	Unknown	[28]
**OX40L**	*In vitro*	Human	Upregulation	BMDCs	Unknown	[44]
**TWEAK**	*In vivo*	Mouse	Upregulation	Brain	Unknown	[45]
**LIGHT**	*In vitro*	Human	Upregulation	A172	Unknown	[46]
	In vivo	Mouse	Upregulation	Lung/brain	Unknown	[23]
**BAFF**	*In vivo*	Mouse	-	-	Not essential to control virus replication at early stages of WNV infection, but they are critical for viral clearance from sera, spleen and brain	[47]
			Upregulation	Neutrophils/ DCs	BAFF from DCs, not neutrophils, is required for protective immunity to WNV	[48]

Cell Lines: H36.12j; *Mus musculus* macrophage cell line ATCC CRL-2449; Renca *Mus musculus* Kidney epithelial cells line ATCC CRL-2947; ARPE-19 : *Homo sapiens* Retinal pigment epithelium cell line CRL-2302; L929 : *Mus musculus* fibroblastic cell line ATCC CCL-1; LAN-2: *Mus musculus* neuroblastoma cell line (RRID:CVCL_1829); U-937: *Homo sapiens* Lymphoma cell line CRL-1593.2; U373: *Homo sapiens* glioblastoma astrocytoma cell-line; SK-N-SH *Homo sapiens* neuroblastoma cell line ATCC HTB-11; THP-1: Monocytic leukemia cells ATCC TIB-202.

Abbreviations: BAFF: B-cell activating factor; BBB: Blood-brain barrier; BMDCs: Bone marrow-derived dendritic cells; BMECs: Brain microvasculature endothelial cells; BMECs: Brain microvascular endothelial cells; CNS: Central nervous system; DCs: Dendritic cells; RPE: retinal pigment epithelium (RPE), IFN: Interferon, IRF: IFN regulatory factor 3; MΦ: Macrophages; NF-κB: Nuclear factor Kappa B; NHP: Non-Human primate; PBMCs: Peripheral blood mononuclear cells; SARM: Sterile alpha and HEAT/Armadillo motif; TLR: Toll-like receptor 3; TRAIL: TNF-related apoptosis-inducing ligand; TWEAK: TNF-related weak inducer of apoptosis.

References:

1. Cheeran, M.C.J.; Hu, S.; Sheng, W.S.; Rashid, A.; Peterson, P.K.; Lokensgard, J.R. Differential Responses of Human Brain Cells to West Nile Virus Infection. *Journal of NeuroVirology* **2005**, *11*, 512–524, doi:10.1080/13550280500384982.

2. Riccetti, S.; Sinigaglia, A.; Desole, G.; Nowotny, N.; Trevisan, M.; Barzon, L. Modelling West Nile Virus and Usutu Virus Pathogenicity in Human Neural Stem Cells. *Viruses* **2020**, *12*, 882, doi:10.3390/v12080882.

3. García-Nicolás, O.; Lewandowska, M.; Ricklin, M.E.; Summerfield, A. Monocyte-Derived Dendritic Cells as Model to Evaluate Species Tropism of Mosquito-Borne Flaviviruses. *Frontiers in Cellular and Infection Microbiology* **2019**, *9*, 5, doi:10.3389/fcimb.2019.00163.

4. Kumar, M.; Verma, S.; Nerurkar, V.R. Pro-Inflammatory Cytokines Derived from West Nile Virus (WNV)-Infected SK-N-SH Cells Mediate Neuroinflammatory Markers and Neuronal Death. *Journal of Neuroinflammation* **2010**, *7*, 73, doi:10.1186/1742-2094-7-73.

5. Yeung, A.W.S.; Wu, W.; Freewan, M.; Stocker, R.; King, N.J.C.; Thomas, S.R. Flavivirus Infection Induces Indoleamine Macrophages via Tumor Necrosis Factor and NF-Kappa B. *Journal ofLeukocyte Biology* **2012**, *91*, 657–666, doi:10.1189/jlb.1011532.

6. Martina, B.E.E.; Koraka, P.; Doel, P. van den; Rimmelzwaan, G.F.; Haagmans, B.L.; Osterhaus, A.D.M.E. DC-SIGN Enhances Infection of Cells with Glycosylated West Nile Virus in Vitro and Virus Replication in Human Dendritic Cells Induces Production of IFN-α and TNF-α. *Virus Research* **2008**, *135*, 64–71, doi:10.1016/j.virusres.2008.02.008.

7. Munoz-erazo, L.; Natoli, R.; Provis, J.M.; Madigan, M.C.; Jonathan, N.; King, C. Microarray Analysis of Gene Expression in West Nile Virus – Infected Human Retinal Pigment Epithelium. *Molecular Vision* **2012**, *18*, 730–743.

8. Qian, F.; Chung, L.; Zheng, W.; Bruno, V.; Alexander, R.P.; Wang, Z.; Wang, X.; Kurscheid, S.; Zhao, H.; Fikrig, E.; et al. Identification of Genes Critical for Resistance to Infection by West Nile Virus Using RNA-Seq Analysis. *Viruses* **2013**, *5*, 1664–1681, doi:10.3390/v5071664.

9. Garcia, M.; Alout, H.; Diop, F.; Damour, A.; Bengue, M.; Weill, M.; Missé, D.; Lévêque, N.; Bodet, C. Innate Immune Response of Primary Human Keratinocytes to West Nile Virus Infection and Its Modulation by Mosquito Saliva. *Front Cell Infect Microbiol* **2018**, *8*, 387, doi:10.3389/fcimb.2018.00387.

10. Xie, G.; Luo, H.; Tian, B.; Mann, B.; Bao, X.; Mcbride, J.; Tesh, R.; Barrett, A.D.; Wang, T. A West Nile Virus NS4B-P38G Mutant Strain Induces Cell Intrinsic Innate Cytokine Responses in Human Monocytic and Macrophage Cells. *Vaccine* **2015**, *33*, 869–878, doi:10.1016/j.vaccine.2014.12.056.A.

11. Aguilar-valenzuela, R.; Netland, J.; Seo, Y.; Bevan, M.J.; Grakoui, A. Dynamics of Tissue-Specific CD8+ T Cell Responses during West Nile Virus Infection. *J Virol* **2018**, *92*, e00014-18, doi:10.1128/JVI.00014-18.

12. Zhang, B.; Patel, J.; Croyle, M.; Diamond, M.S.; Klein, R.S. TNF-α-Dependent Regulation of CXCR3 Expression Modulates Neuronal Survival during West Nile Virus Encephalitis. *Journal of Neuroimmunology* **2010**, *224*, 28–38, doi:10.1016/j.jneuroim.2010.05.003.

13. Shrestha, B.; Zhang, B.; Purtha, W.E.; Klein, R.S.; Diamond, M.S. Tumor Necrosis Factor Alpha Protects against Lethal West Nile Virus Infection by Promoting Trafficking of Mononuclear Leukocytes into the Central Nervous System. *Journal of Virology* **2008**, *82*, 8956–8964, doi:10.1128/jvi.01118-08.

14. Cheng, Y.; King, N.J.C.; Kesson, A.M. The Role of Tumor Necrosis Factor in Modulating Responses of Murine Embryo Fibroblasts by Flavivirus , West Nile B. *Virology* **2004**, *329*, 361–370, doi:10.1016/j.virol.2004.06.050.

15. Saxenaa, V.; Weltea, T.; Baob, X.; Xiea, G.; Wanga, J.; Higgsc, S.; Teshd, R.B.; Tian Wanga A Hamster-Derived West Nile Virus Strain Is Highly Attenuated and Induces a Differential Proinflammatory Cytokine Response in Two Murine Cell Lines. *Virus Res.* **2013**, *167*, 179–187, doi:10.1016/j.virusres.2012.04.013.A.

16. Fang, H.; Welte, T.; Zheng, X.; Chang, G.-J.J.; Holbrook, M.R.; Soong, L.; Tian Wang Γδ T Cells Promote the Maturation of Dendritic Cells During West Nile Virus Infection. *FEMS Immunol Med Microbiol.* **2010**, *59*, 71–80, doi:10.1111/j.1574-695X.2010.00663.x.

17. Luo, H.; Winkelmann, E.R.; Zhu, S.; Ru, W.; Mays, E.; Silvas, J.A.; Vollmer, L.L.; Gao, J.; Peng, B.H.; Bopp, N.E.; et al. Peli1 Facilitates Virus Replication and Promotes Neuroinflammation during West Nile Virus Infection. *Journal of Clinical Investigation* **2018**, *128*, 4980–4991, doi:10.1172/JCI99902.

18. Sapkal, G.N.; Harini, S.; Ayachit, V.M.; Fulmali, P. v; Mahamuni, S.A.; Bondre, V.P.; Gore, M.M. Neutralization Escape Variant of West Nile Virus Associated with Altered Peripheral Pathogenicity and Differential Cytokine Profile. *Virus Research* **2011**, *158*, 130–139, doi:10.1016/j.virusres.2011.03.023.

19. Shirato, K.; Miyoshi, H.; Kariwa, H.; Takashima, I. The Kinetics of Proinflammatory Cytokines in Murine Peritoneal Macrophages Infected with Envelope Protein-Glycosylated or Non-Glycosylated West Nile Virus. *Virus Research* **2006**, *121*, 11–16, doi:10.1016/j.virusres.2006.03.010.

20. Wang, T.; Town, T.; Alexopoulou, L.; Anderson, J.F.; Fikrig, E.; Flavell, R.A. Toll-like Receptor 3 Mediates West Nile Virus Entry into the Brain Causing Lethal Encephalitis. *Nature Medicine* **2004**, *10*, 1366–1373, doi:10.1038/nm1140.

21. Xiea, G.; Weltea, T.; Wanga, J.; Whitemanb, M.C.; Wickerb, J.A.; Saxenaa, V.; Conga, Y.; Barretta, A.D.T.; Wang, T. A West Nile Virus NS4B-P38G Mutant Strain Induces Adaptive Immunity via TLR7-MyD88-Dependent and Independent Signaling Pathways. *Vaccine* **2013**, *31*, 4143–4151, doi:10.1016/j.vaccine.2013.06.093.A.

22. Suthar, M.S.; Brassil, M.M.; Blahnik, G.; McMillan, A.; Ramos, H.J.; Proll, S.C.; Belisle, S.E.; Katze, M.G.; Gale, M. A Systems Biology Approach Reveals That Tissue Tropism to West Nile Virus Is Regulated by Antiviral Genes and Innate Immune Cellular Processes. *PLoS Pathogens* **2013**, *9*, e1003168, doi:10.1371/journal.ppat.1003168.

23. Peña, J.; Plante, J.A.; Carillo, A.C.; Roberts, K.K.; Smith, J.K.; Juelich, T.L.; Beasley, D.W.C.; Freiberg, A.N.; Labute, M.X.; Naraghi-Arani, P. Multiplexed Digital MRNA Profiling of the Inflammatory Response in the West Nile Swiss Webster Mouse Model. *PLoS Neglected Tropical Diseases* **2014**, *8*, e3216, doi:10.1371/journal.pntd.0003216.

24. Kumar, M.; Roe, K.; O'Connell, M.; Nerurkar, V.R. Induction of Virus-Specific Effector Immune Cell Response Limits Virus Replication and Severe Disease in Mice Infected with Non-Lethal West Nile Virus Eg101 Strain. *Journal of Neuroinflammation* **2015**, *12*, 178, doi:10.1186/s12974-015-0400-y.

25. Szretter, K.J.; Samuel, M.A.; Gilfillan, S.; Fuchs, A.; Colonna, M.; Diamond, M.S. The Immune Adaptor Molecule SARM Modulates Tumor Necrosis Factor Alpha Production and Microglia Activation in the Brainstem and Restricts West Nile Virus Pathogenesis. *Journal of Virology* **2009**, *83*, 9329–9338, doi:10.1128/jvi.00836-09.

26. Garcia-tapia, D.; Hassett, D.E.; Mitchell Jr, W.J.; Johnson, G.C.; Kleiboeker, S.B. West Nile Virus Encephalitis : Sequential Histopathological and Immunological Events in a Murine Model of Infection. *Journal of NeuroVirology* **2007**, *13*, 130–138, doi:10.1080/13550280601187185.

27. Glass, W.G.; Lim, J.K.; Cholera, R.; Pletnev, A.G.; Gao, J.L.; Murphy, P.M. Chemokine Receptor CCR5 Promotes Leukocyte Trafficking to the Brain and Survival in West Nile Virus Infection. *Journal of Experimental Medicine* **2005**, *202*, 1087–1098, doi:10.1084/jem.20042530.

28. Kumar, M.; Nerurkar, V.R. Integrated Analysis of MicroRNAs and Their Disease Related Targets in the Brain of Mice Infected with West Nile Virus. *Virology* **2014**, *0*, 143–151, doi:10.1016/j.virol.2014.01.004.

29. Lim, S.M.; van den Ham, H.J.; Oduber, M.; Martina, E.; Zaaraoui-Boutahar, F.; Roose, J.M.; van IJcken, W.F.J.; Osterhaus, A.D.M.E.; Andeweg, A.C.; Koraka, P.; et al. Transcriptomic Analyses Reveal Differential Gene Expression of Immune and Cell Death Pathways in the Brains of Mice Infected with West Nile Virus and Chikungunya Virus. *Frontiers in Microbiology* **2017**, *8*, 1556, doi:10.3389/fmicb.2017.01556.

30. Getts, D.R.; Matsumoto, I.; Mu, M.; Getts, T.; Radford, J.; Shrestha, B.; Campbell, I.L.; King, N.J.C. Role of IFN-γ in an Experimental Murine Model of West Nile Virus-Induced Seizures. *J of Neurochemistry* **2007**, *103*, 1019–1030, doi:10.1111/j.1471-4159.2007.04798.x.

31. Shrestha, B.; Wang, T.; Samuel, M.A.; Whitby, K.; Craft, J.; Fikrig, E.; Diamond, M.S. Gamma Interferon Plays a Crucial Early Antiviral Role in Protection against West Nile Virus Infection. *Journal of Virology* **2006**, *80*, 5338–5348, doi:10.1128/jvi.00274-06.

32. Byrne, S.N.; Halliday, G.M.; Johnston, L.J.; King, N.J.C. Interleukin-1β but Not Tumor Necrosis Factor Is Involved in West Nile Virus-Induced Langerhans Cell Migration from the Skin in C57BL/6 Mice. *Journal of Investigative Dermatology* **2001**, *117*, 702–709, doi:10.1046/j.0022-202x.2001.01454.x.

33. Kumar, M.; Belcaid, M.; Nerurkar, V.R. Identification of Host Genes Leading to West Nile Virus Encephalitis in Mice Brain Using RNA-Seq Analysis. *Scientific Reports* **2016**, *6*, 26350, doi:10.1038/srep26350.

34. Uddin, M.J.; Suen, W.W.; Prow, N.A.; Hall, R.A.; Bielefeldt-Ohmann, H. West Nile Virus Challenge Alters the Transcription Profiles of Innate Immune Genes in Rabbit Peripheral Blood Mononuclear Cells. *Frontiers in Veterinary Science* **2015**, *2*, 76, doi:10.3389/fvets.2015.00076.

35. Suen, W.W.; Uddin, M.J.; Prow, N.A.; Bowen, R.A.; Hall, R.A.; Bielefeldt-Ohmann, H. Tissue-Specific Transcription Profile of Cytokine and Chemokine Genes Associated with Flavivirus Control and Non-Lethal Neuropathogenesis in Rabbits. *Virology* **2016**, *494*, 1–14, doi:10.1016/j.virol.2016.03.026.

36. Bielefeldt-ohmann, H.; Bosco-lauth, A.; Hartwig, A.; Uddin, M.J.; Barcelon, J.; Suen, W.W.; Wang, W.; Hall, R.A.; Bowen, R.A. Microbial Pathogenesis Characterization of Non-Lethal West Nile Virus (WNV) Infection in Horses : Subclinical Pathology and Innate Immune Response. *Microbial Pathogenesis* **2017**, *103*, 71–79, doi:10.1016/j.micpath.2016.12.018.

37. Maximova, O.A.; Sturdevant, D.E.; Kash, J.C.; Kanakabandi, K.; Xiao, Y.; Minai, M.; Moore, I.N.; Taubenberger, J.; Martens, C.; Cohen, J.I.; et al. Virus Infection of the Cns Disrupts the Immune-Neural-Synaptic Axis via Induction of Pleiotropic Gene Regulation of Host Responses. *Elife* **2021**, *10*, e62273, doi:10.7554/eLife.62273.

38. Quick, E.D.; Leser, J.S.; Clarke, P.; Tyler, K.L. Activation of Intrinsic Immune Responses and Microglial Phagocytosis in an Ex Vivo Spinal Cord Slice Culture Model of West Nile Virus Infection. *Journal of Virology* **2014**, *88*, 13005–13014, doi:10.1128/jvi.01994-14.

39. Shrestha, B.; Pinto, A.K.; Green, S.; Bosch, I.; Diamond, M.S. CD8+ T Cells Use TRAIL To Restrict West Nile Virus Pathogenesis by Controlling Infection in Neurons. *Journal of Virology* **2012**, *86*, 8937–8948, doi:10.1128/jvi.00673-12.

40. O'Neal, J.T.; Upadhyay, A.A.; Wolabaugh, A.; Patel, N.B.; Bosinger, S.E.; Suthar, M.S. West Nile Virus-Inclusive Single-Cell RNA Sequencing Reveals Heterogeneity in the Type I Interferon Response within Single Cells. *J Virol* **2019**, *93*, e01778-18, doi:10.1128/JVI.01778-18.

41. Shrestha, B.; Diamond, M.S. Fas Ligand Interactions Contribute to CD8 + T-Cell-Mediated Control of West Nile Virus Infection in the Central Nervous System . *Journal of Virology* **2007**, *81*, 11749–11757, doi:10.1128/jvi.01136-07.

42. Aarreberg, L.D.; Wilkins, C.; Ramos, H.J.; Green, R.; Davis, M.A.; Chow, K.; Gale, M. Interleukin-1 β Signaling in Dendritic Cells Induces Antiviral Interferon Responses. *mBio* **2018**, *9*, e00342-18, doi:10.1128/mBio .00342-18.

43. Sandip, S.; Lal, G. Role of Tumor Necrosis Factor Superfamily in Neuroinflammation and Autoimmunity. *Front. Immunol* **2015**, *6*, 364, doi:10.3389/fimmu.2015.00364.

44. Kovats, S.; Turner, S.; Simmons, A.; Powe, T.; Chakravarty, E.; Alberola-Ila, J. West Nile Virus-Infected Human Dendritic Cells Fail to Fully Activate Invariant Natural Killer T Cells. *Clinical and Experimental Immunology* **2016**, *186*, 214–226, doi:10.1111/cei.12850.

45. Clarke, P.; Leser, J.S.; Bowen, R.A.; Tylera, K.L. Virus-Induced Transcriptional Changes in the Brain Include the Differential Expression of Genes Associated with Interferon, Apoptosis, Interleukin 17 Receptor A, and Glutamate Signaling as Well as Flavivirus-Specific Upregulation of TRNA Synthetases. *mBio* **2014**, *5*, e00902-14, doi:10.1128/mBio.00902-14.

46. Koh, W.L.; Ng, M.L. Molecular Mechanisms of West Nile Virus Pathogenesis in Brain Cells. *Emerging Infectious Diseases* **2005**, *11*, 629–632, doi:10.3201/eid1104.041076.

47. Giordano, D.; Draves, K.E.; Young, L.B.; Roe, K.; Bryan, M.A.; Dresch, C.; Richner, J.M.; Diamond, M.S.; Gale, M.; Clark, E.A. Protection of Mice Deficient in Mature B Cells from West Nile Virus Infection by Passive and Active Immunization. *PLoS Pathog* **2017**, *13*, e1006743, doi:10.1371/journal.ppat.1006743.

48. Giordano, D.; Kuley, R.; Draves, K.E.; Roe, K.; Holder, U.; Giltiay, V.; Clark, E.A. B Cell Activating Factor (BAFF) Produced by Neutrophils and Dendritic Cells Is Regulated Differently and Has Distinct Roles in Ab Responses and Protective Immunity against West Nile Virus. *J Immunol.* **2020**, *204*, 1508–1520, doi:10.4049/jimmunol.1901120.B.
